# Acute exercise and oxidative stress: a 30 year history

**DOI:** 10.1186/1476-5918-8-1

**Published:** 2009-01-13

**Authors:** Kelsey Fisher-Wellman, Richard J Bloomer

**Affiliations:** 1 Cardiorespiratory/Metabolic Laboratory, Department of Health and Sport Sciences, The University of Memphis, 161F Elma Neal Roane Fieldhouse, Memphis, TN 38152, USA

## Abstract

The topic of exercise-induced oxidative stress has received considerable attention in recent years, with close to 300 original investigations published since the early work of Dillard and colleagues in 1978. Single bouts of aerobic and anaerobic exercise can induce an acute state of oxidative stress. This is indicated by an increased presence of oxidized molecules in a variety of tissues. Exercise mode, intensity, and duration, as well as the subject population tested, all can impact the extent of oxidation. Moreover, the use of antioxidant supplements can impact the findings. Although a single bout of exercise often leads to an acute oxidative stress, in accordance with the principle of hormesis, such an increase appears necessary to allow for an up-regulation in endogenous antioxidant defenses. This review presents a comprehensive summary of original investigations focused on exercise-induced oxidative stress. This should provide the reader with a well-documented account of the research done within this area of science over the past 30 years.

## Background

Oxidative stress is a condition in which the delicate balance existing between prooxidant (free radicals) production and their subsequent amelioration via the antioxidant defense system becomes skewed in favor of free radical expression [1]. The production or formation of free radicals in vivo is primarily initiated by the consumption of molecular oxygen, which, due to its structure is in fact a radical species itself [1]. A free radical is any species capable of existence, containing one or more unpaired electrons [2]. Although a multitude of free radicals exist [hydrogen atoms, transition metal ions, carbon centered radicals (e.g., trichloromethyl), sulfur centered radicals (e.g., thiyl)] [2], those derived from either oxygen and/or nitrogen represent the most important class of radicals generated in living systems [3,4]. Both the radicals themselves as well as the nonradical species created via interaction with free radicals are collectively referred to as reactive oxygen/nitrogen species (RONS) [5]. The body's antioxidant defense system serves to protect the cells from excess RONS production and is comprised of both endogenous (bilirubin, uric acid, superoxide dismutases, catalase, glutathione peroxidase, etc.) and exogenous (carotenoids, tocopherols, ascorbate, bioflavonoids, etc.) compounds [6]. The exogenous compounds are consumed in the diet and come primarily from ingestion of fruits and vegetables [7].

It is clear that a basal level of RONS production and removal is constantly occurring, in turn eliciting both positive and negative effects on physiological function. In living systems, this delicate balance eluded to above (free radical production vs. antioxidant defense) serves to determine the intracellular redox state [8], which in turn plays a role in optimizing cellular function. The redox state and/or redox balance is representative of the oxidation/reduction potential present within the cell and is tightly regulated similar to that of pH, and is commonly assessed via the ratio between reduced (GSH) and oxidized (GSSG) glutathione (the major non-enzymatic antioxidant) or other thiol/disulfide compounds [5]. Mammalian cells are endowed with signaling pathways that are sensitive to the intracellular redox environment and can be activated by oxidative stress [9]. Thus, transient disturbances in redox balance, causing a shift towards a more oxidizing environment, can occur via increased RONS production and/or decreased antioxidant defense and appear to serve as a "signal" for the activation of several cell signaling mechanisms important for optimal physiological function [10]. Examples of specific redox-sensitive regulated functions and their associated signaling mechanism include, but are not limited to: 1) regulation of vascular tone via activation of guanylate cyclase [11] or the transcriptional/posttranscriptional regulation of nitric oxide synthase (NOS) via activation of nuclear factor κB (NF-κB) or mitogen-activated protein kinases (MAPK) [12]; 2) Amplification of immune responses and apoptosis via activation of activator protein 1 (AP-1) and NF-κB transcription factors in human T cells [13,14]; 3) Regulation of insulin receptor kinase activity via increased activity of protein tyrosine phosphotases [15]; and 4) Increased expression of antioxidant enzymes and/or glutathione in response to MAPK and NF-κB activation in an effort to restore redox balance [9]. The latter example is particularly applicable to exercise, as an increase in RONS during and following acute exercise is believed to serve as the necessary "signal" for the hormetic-associated upregulation in antioxidant defense commonly observed with chronic exercise training, and will be discussed further later in this review. The above examples are offered in an effort to provide a brief overview of the importance of RONS in physiological function. However, a thorough discussion of the role of RONS in gene expression and cellular control is beyond the scope of this review. For more information the reader is referred to a few excellent reviews within the area [8-10,16].

While a shift in the redox state in favor of RONS expression is indeed needed to initiate such signaling pathways, execution of such signals are contingent upon a return to reducing conditions [10]. Therefore, conditions that favor accelerated and/or chronic production of RONS may serve to overwhelm the capacity of the antioxidant defense system in place, thereby disrupting normal redox-sensitive signaling and causing a permanent shift in redox balance [10]. Moreover, this permanent shift in the redox environment could then induce damaging effects via direct RONS-mediated oxidative damage to nucleic acids, lipids and proteins [17], as well as through changes in gene expression that promote apoptosis within healthy cells, and systemic inflammation [16]. Both moderate and excessive shifts in redox potential, resulting from chronic oxidative stress have been suggested to play a role in the functional decline commonly observed with aging, as well as in the pathophysiology of several diseased states, respectively [10,16]. In fact, oxidative stress has been suggested to play a primary or secondary role in the development of multiple (> 100) acute and chronic human diseases [17]. To summarize, RONS are not inherently harmful; however, in response to chronic exposure to excessive and/or ectopic production of RONS, the system can become unbalanced (free radicals > defenses), potentially resulting in a shift in the intracellular redox balance towards a more oxidizing environment, in turn promoting oxidative damage, inflammation, ill-health, and disease.

**Figure 1:**
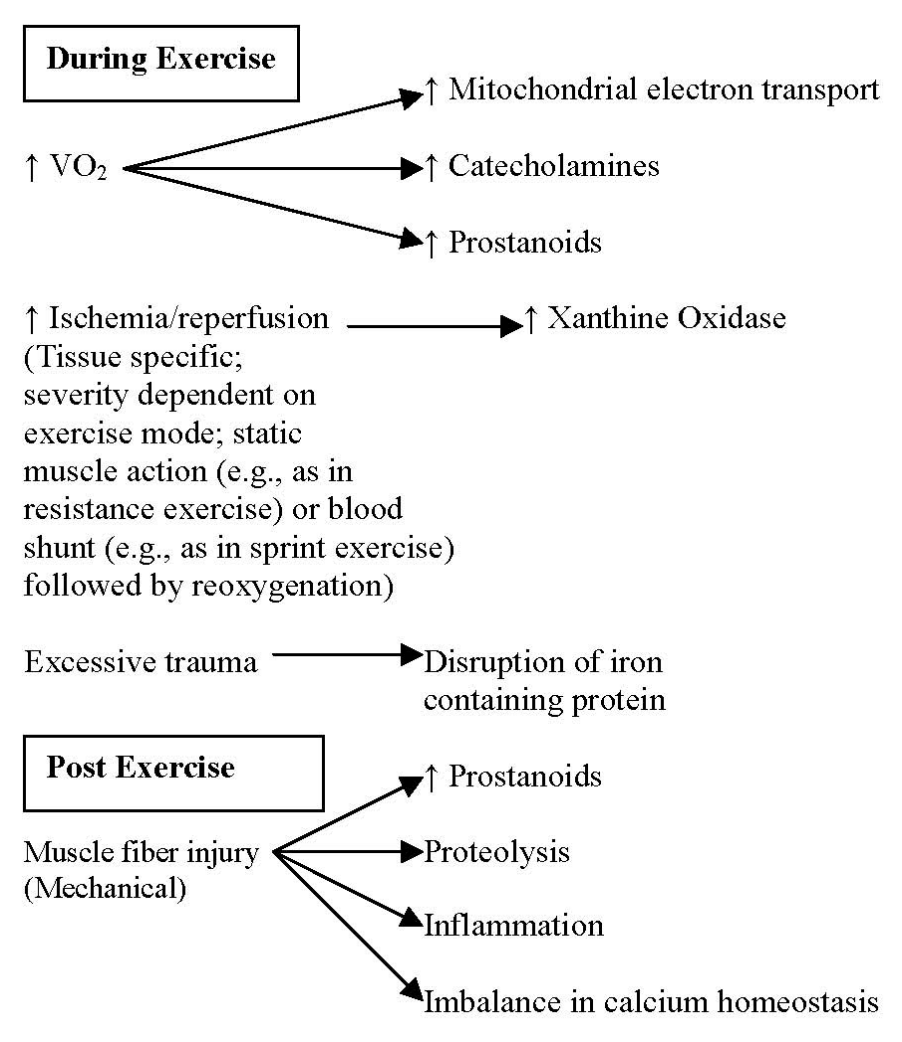
**Potential mechanisms of increased RONS production related to an acute bout of exercise**. Adapted with permission from Bloomer RJ, & Goldfarb AH. Anaerobic exercise and oxidative stress: A review. *Canadian Journal of Applied Physiology*, 29(3): 245–263, 2004.

Overproduction of RONS can result from a variety of stressors, such as exposure to environmental pollutants [2], excessive nutrient intake [18], or physical exercise [19]. However, simply stated, any situation in which the consumption of oxygen is increased, as during physical exercise, could result in an acute state of oxidative stress. Primary RONS generation in response to acute exercise can occur via several pathways. These include mitochondrial respiration (electron leakage from electron transport chain and subsequent production of the superoxide radical), prostanoid metabolism, the autooxidation of catecholoamines, and oxidase enzymatic activity (NAD(P)H oxidase, xanthine oxidase) [20]. The initial increase in RONS during exercise, as well as following cessation of the work bout can lead to additional secondary generation of prooxidants via phagocytic respiratory burst, a loss of calcium homeostasis and/or the destruction of iron-containing proteins [20]. Moreover, while the pathways listed above represent potential sources of RONS during exercise, specific RONS generation likely depends on the mode (aerobic, anaerobic), intensity, and duration of exercise, as varying types of exercise differ in their respective energy requirements, levels of oxygen consumption, and mechanical stresses imposed on the tissues [20]. These potential sites of RONS generation during exercise can be viewed in Figure 1.

Since the initial finding of increased lipid peroxidation following acute aerobic exercise in 1978 [21], the field of oxidative stress and exercise has expanded substantially, evident by the numerous original investigations conducted over the past 30 years. This increased interest is fueled by several factors, including the enhanced awareness of the role of RONS in human disease, a greater effort to promote exercise as a means for the improvement and/or maintenance of health, as well as the widespread development and availability of various antioxidant agents (of which efficacy is often tested using exercise as a stimulus of RONS). Although much of the early work has viewed exercise-induced RONS production as a potential detriment to physiological function (i.e., decreased performance and immune function, and increased fatigue), more recent work is investigating an alternative role for RONS production in regards to favorable exercise-induced adaptations.

Much of the advances in the field have been made possible by substantial improvements in measurement techniques over the past 30 years, as well as the fact that many analytical tools needed for this work are more user-friendly and readily available than ever before. Since the initial discoveries of Dilliard and colleagues [21], several commercial assay kits have been made available for the measurement of oxidative stress, with many new kits emerging each year. Furthermore, the discovery and utilization of F_2_-isoprostanes, a prostaglandin like compound, measured via gas chromotomography mass spectrometry has emerged as a substantially more reliable and valid measure of lipid peroxidation [22]. Newly developed ELISA kits for both isoprostanes as well as protein carbonyls are also now available, proving an opportunity for a more widespread use of these biomarkers.

In regards to measurement of oxidative stress, due to the high reactivity and relatively short half lives (e.g., 10^-5^, 10^-9 ^seconds for superoxide radical and hydroxyl radical, respectively) of RONS, direct measurement is extremely difficult to employ. However, direct assessment of free radical production is possible via electron spin resonance spectroscopy (ESR) involving spin traps, as well as two other less common techniques such as radiolysis and laser flash photolysis [23]. ESR works by recording the energy changes that occur as unpaired electrons align in response to a magnetic field [1]. Due to the high cost of such equipment and the high degree of labor associated with each direct method, the majority of free radial research related to exercise has utilized indirect methods for the assessment of resultant oxidative stress.

Indirect assessment of oxidative stress involves the measurement of the more stable molecular products formed via the reaction of RONS with certain biomolecules. Common molecular products include stable metabolites (e.g., nitrate/nitrite), and/or concentrations of oxidation target products, including lipid peroxidation end products [isoprostanes, malondialdehyde (MDA), thiobarbituric acid reactive substances (TBARS), lipid hydroperoxides (LOOH), conjugated dienes (CD), oxidized low density lipoprotein (oxLDL)], oxidized proteins [protein carbonyls (PC), individual oxidized amino acids, nitrotyrosine (NT)], and nucleic acids [8-hydroxy-2-deoxyguanosine (8-OHdG), oxidized DNA bases (via the Comet Assay), strand breaks] [17]. Additionally, oxidative stress can be measured by observing alterations in the body's antioxidant defense system. This is typically done by measuring the redox changes in the major endogenous antioxidant glutathione, as well as circulating levels of vitamin E, and vitamin C. Moreover, the activity of certain antioxidant enzymes [e.g., superoxide dismutase (SOD), glutathione peroxidase (GPx), catalase (CAT), glutathione reductase (GR)] can be assessed as indicators of the oxidative stress imposed on the tissue. Numerous antioxidant capacity assays also exist and include: Trolox Equivalent Antioxidant Capacity (TEAC), Total Antioxidant Status (TAS), Ferric Reducing Ability of Plasma (FRAP), Total Radical-Trapping Antioxidant Parameter (TRAP), and Oxygen Radical Absorbance Capacity (ORAC).

Evidence for increased RONS production during and following exercise is provided by numerous investigations noting an increase in various oxidative stress biomarkers following both acute aerobic (for review, see [19]) and anaerobic exercise (for review, see [24]). In addition, direct measurement of free radical production via electron spin resonance following acute exercise in animals [25] and humans [26-30] has also been reported.

From work over the past three decades, it is clear that exercise of sufficient volume, intensity, and duration can lead to an increase in RONS production, which may lead to the oxidation of several biological molecules (lipids, proteins, nucleic acids). Whether or not this condition is indicative of a harmful stimulus however, remains a topic of debate [19,31]. That is, due to the potential role of RONS in impairing exercise performance via altering contractile function and/or accelerating muscle damage/fatigue (secondary to the oxidation of contractile and/or mitochondrial enzymes) [32-34], coupled with their association with human disease [17], exercise-induced RONS have commonly been viewed as a detriment to physiological function. Hence, methods to reduce radical production and subsequent oxidative damage during and following physical exercise have been a priority of much research activity. While excessive prooxidant production, arising from any form of extreme aerobic or anaerobic exercise (i.e., marathon, aerobic/anaerobic overtraining) may have the potential to result in significant cellular disruption, there presently exist no "cause and effect" data to indicate that such an increase in RONS resulting from acute exercise actually causes ill-health and disease. To the contrary, and in accordance with the principle of hormesis, a low grade oxidative stress appears necessary for various physiological adaptations [35-37]. Such a repeated exposure of the system to increased RONS production from chronic exercise training leads to an upregulation in the body's antioxidant defense system [38,39] and associated shift in redox balance in favor of a more reducing environment, thus providing adaptive protection from RONS during subsequent training sessions, as well as when exposed to non-exercise related conditions. Taken together, exercise-induced oxidative stress may operate in a similar fashion to all other principles of exercise science. That is, in order for an adaptation to occur (e.g., increased antioxidant defense, hypertrophy, strength), the physiological stimulus applied (in this case RONS production) must exceed a certain minimal threshold, effectively overloading the system. If overload is achieved, the physiological capacity of the body will expand or adapt; ultimately leading to improvements in health and/or human performance.

This review is intended to provide a comprehensive summary of original investigations focused on exercise-induced oxidative stress over the past 30 years. It presents data from close to 300 original investigations separated by aerobic and anaerobic exercise modes. Detailed tables inclusive of the tissues studied and individual times of measurement for each sample are provided (see Additional file 1). In an attempt to identify the relevant literature, a comprehensive search was performed using PubMed and Google Scholar. The following search terms were included in multiple combinations: oxidative stress and exercise, oxidative stress and aerobic exercise, oxidative stress and anaerobic exercise, oxidative stress and resistance exercise. Further PubMed searching was performed by selecting the "See all related articles" function, thus providing an additional extensive list of publications. Further searching was performed by manual scanning of the reference lists of several review articles, as well as original investigations. The search was conducted between October and December 2007. Although we believe to have identified the bulk of original investigations within this area by using the above techniques, admittedly, some investigations may have escaped our search and are therefore not included. We apologize to those authors whose work is not cited here.

### Overview/limitations of oxidative stress and acute exercise research

Prior to the discussion of the collective results of the relative studies, it is imperative to understand some basic limitations of research in the area of oxidative stress and acute exercise. The multiple body systems, inclusive of the antioxidant defense system, function in a complex and vastly interconnected fashion. Therefore, concrete conclusions regarding precisely how and why RONS are produced during exercise, remains a topic of continued study. To claim a complete understanding of these processes at this time may largely underestimate the complexity of the human body and associated redox systems. This simply means that current understandings and findings relative to RONS and acute exercise should remain open to further interpretation and discovery. Of course, a key element involved in the progression of a given scientific area is a clear understanding and familiarization with current findings and beliefs. It is the intent of this review to provide such information.

Currently, it is clear that both acute aerobic [25-27] and anaerobic [28-30] exercise has the potential to result in increased free radical production, which may or may not result in acute oxidative stress. As stated earlier, in order for oxidative stress to occur, the RONS produced during exercise must exceed the antioxidant defense system present, thereby resulting in oxidative damage to specific biomolecules [40]. Different exercise protocols may induce varying levels of RONS production, as oxidative damage has been shown to be both intensity [41,42] and duration [43] dependent. During low-intensity and duration protocols, antioxidant defenses appear sufficient to meet the RONS production, but as intensity and/or duration of exercise increases, these defenses are no longer adequate, potentially resulting in oxidative damage to surrounding tissues [44]. Other factors appear to impact the degree of antioxidant defenses present, including age [45], training status [38,39], and dietary intake [7]. If oxidative stress does occur, detection depends to a large degree on the tissue sampled, the timing of a given sample, as well as the sensitivity and specificity of the biomarker chosen [17]. Significant or null findings may be related to the lack of specificity of the chosen biomarker (as has been suggested for TBARS [46]), improper sampling protocol (too few measures or too short time course), or improper tissue (blood or urine vs. skeletal muscle). Under these circumstances, it is possible that in investigations where oxidative stress was not observed following acute exercise, oxidative stress may have occurred prior to or after sample collection or in tissue (e.g., skeletal muscle, cardiac, liver, brain) other than that which was sampled (most commonly blood). Taken together, it appears that several factors influence both the onset of oxidative stress (intensity and duration of exercise, age, training status and dietary intake of subjects) as well as the detection of such stress in vivo (biomarker chosen, tissue sampled, timing of sampling). These variables may partially explain some of the inconsistency present within the literature.

### Acute aerobic exercise: human studies

The majority of research in the area of oxidative stress and acute exercise in humans has utilized aerobic exercise protocols (> 160 original investigations). Typical protocols have included submaximal or maximal effort aerobic exercise either on a treadmill or cycle ergometer, with the majority of investigations utilizing a graded exercise test (GXT) to induce an oxidant stress. Most laboratory based protocols have involved short to moderate duration exercise bouts (≤ 2 hours), while a few laboratory protocols, and the more common "field" tests, have included much longer times of exercise (> 2 hours). In addition, some treadmill studies have focused on downhill running, involving eccentric bias in order to induce muscle injury. For the purpose of this review, as a means of classification, all exercise protocols discussed in the text will be referred to as maximal or submaximal, as detailing each specific protocol would not be practical due to the variation within each study design. Results will be discussed relative to each specific biomarker utilized, with the initial section providing a brief illustration of the nature of each biomarker that can be referred to throughout for clarification purposes. Studies involving non-eccentric aerobic exercise without antioxidant supplementation will be discussed below and can be viewed in Table 1 of Additional file 1.

### Short to moderate duration protocols

#### Lipid peroxidation

The most common method utilized to indicate exercise induced oxidative damage in regards to non-eccentric aerobic exercise has been the assessment of lipid peroxidation, with malondialdehyde (MDA) and thiobarbituric acid reactive substances (TBARS) representing the most commonly used assays. Malondialdehyde is a three carbon chain aldehyde produced during decomposition of a lipid hydroperoxide. Additionally, thiobarbituric acid reactive substances (TBARS) is an assay used to measure aldehyde products (primarily MDA) formed via decomposition of lipid hydroperoxides. However, the TBARS assay lacks specificity, for in addition to aldehydes, TBA also can react with several other biological molecules (such as carbohydrates, sialic acid, or prostaglandins), thus interfering with the assay [46]. Further evidence for the lack of specificity of the assay is evident by the fact that the majority of authors have noted an increase in TBARS following a variety of exercise protocols, whereas null findings appear much more common when measuring MDA or isoprostanes specifically.

Numerous studies have reported an increase in TBARS following both maximal [47-55] and submaximal [56-63] exercise in humans, with values typically returning to baseline within one hour post exercise [48,50], unless maximal exercise is preceded by a submaximal stimuli of sufficient intensity and duration [52]. In opposition to these findings, a few studies have reported no increase in TBARS despite the use of similar maximal [64-67] and submaximal [68-71] protocols.

In regards to the measurement of MDA specifically, an apposing trend is evident, thus drawing further suspicion to the specificity of the TBARS assay. The majority of studies have noted no increase in MDA following maximal [72-81] or submaximal [82-90] exercise, with fewer investigations reporting a significant increase [27,91-100]. However, those studies reporting significant increases typically utilized maximal (GXT) [27,91-96,100] or near maximal (~75%VO_2max_) [97-99] exercise protocols, indicating a role of intensity in MDA formation.

Other markers of lipid peroxidation have included measurement of the susceptibility of LDL cholesterol to undergo oxidation in vitro (reported as a decrease in lag time to oxidation), accumulation of other lipid peroxidation products such as conjugated dienes (CD), and lipid hydroperoxides (LOOH), as well as breath analysis of certain hydrocarbons, such as pentane and ethane. To our knowledge, all investigations involving acute aerobic exercise, when measuring expired hydrocarbons [21,73,97,101], or the susceptibility of LDL cholesterol to undergo oxidation in vitro [94,95,102,103], have noted a unanimous increase. No change [102,104] or an increase [105] has been observed in CD following a GXT. Similar to CD, results regarding measurement of LOOH have been varied, with some studies noting an increase [27,60,79,88,93] or no change [75,104,106-111] post exercise.

In relation to our discussion of lipid peroxidation, it should be noted that F_2_-isoprostanes, a prostaglandin-like compound generated in vivo by non-enzymatic peroxidation of arachidonic acid (an omega-6 fatty acid present in the phospholipids of cell membranes), is regarded as the most reliable approache for the assessment of free radical mediated lipid peroxidation [17]. Although much more involved and time consuming than the above methods, the specificity is much greater. A detailed discussion of the measurement technique for F_2_-isoprostanes has been presented recently by Milne and coworkers [112]. Increased concentrations of F_2_-isoprostanes have been reported by a few investigators [42,113], with increases responding in an intensity dependent manner [42]. Null findings have also been reported [114,115]; however, these results were likely due to a low intensity protocol (50%VO_2max_) [114] or the fact that subjects were considered to be trained athletes [115] and likely "protected" from RONS due to an enhanced endogenous antioxidant defense system.

#### Glutathione

In addition to lipid peroxidation, the measurement of redox changes in glutathione (the major non-enzymatic endogenous antioxidant) has also been routinely performed as a representation of exercise induced oxidative stress. Typically, a decrease in reduced glutathione (GSH) [48-50,52,53,56,58,70,82,86,88,99,106,108,115-120], an increase in oxidized glutathione (GSSG) [52,53,56,58,61,63,70,82,86,99,106,115-117,119-121], with no change to total glutathione concentration (TGSH) [56,61,63,99,106,107,118,120,122] has been reported following a variety of non-eccentric aerobic exercise protocols. Glutathione status typically returns to basal levels within 15–30 minutes of recovery [48,50,106,116]. Studies reporting null findings for glutathione redox status [53,68,107,108,123,124] may be partially related to the timing of sampling, as GSSG is rapidly reduced in vivo by way of glutathione reductase [5], in addition to the trained status of the subjects [108] or an insufficient intensity of exercise [68,107].

#### DNA oxidation

DNA subjected to attack by RONS results in the formation of a variety of base and sugar modification products [125]. The presence of these modified products is used to indicate oxidative stress, as they are not present during normal nucleotide metabolism. Typically, the product 8-hydroxy-2-deoxyguanosine (8-OHdG) has been measured as an index of exercise induced oxidation of DNA. Aside from two investigations noting a significant increase in 8-OHdG [69,83], the majority of studies have reported no change following a variety of exercise protocols [74,82,88,89,99,106,126-129]. Null findings may be partially due to the fact that moderate duration and/or intensity aerobic exercise may not be sufficient to elicit an increase in 8-OHdG [82], possibly due to the rapid repair of DNA following oxidation [130,131], as an increase in the activity of certain DNA repair enzymes has been observed following acute aerobic exercise [131]. Aside from the measurement of 8-OHdG, assessment of DNA damage has also been performed using the single cell gel electrophoresis assay (Comet assay) which detects DNA damage with high sensitivity [72]. In these investigations, increases have been noted in DNA damage post exercise [72,80,132].

#### Protein oxidation

Proteins are major targets for RONS because of their high overall abundance in biological systems and it has been estimated that proteins can scavenge the majority (50–75%) of RONS generated [133]. Oxidative damage to proteins can occur directly by interaction of the protein with RONS or indirectly by interaction of the protein with a secondary product (resulting from interaction of radical with lipid or sugar molecule) [17]. Modification of a protein under conditions of oxidative stress can occur via peptide backbone cleavage, cross-linking, and/or modification of the side chain of virtually every amino acid [17]. Moreover, most protein damage is irreparable and oxidative modification of the protein structure can lead to loss of enzymatic, contractile, or structural function in the affected proteins, thus making them increasingly susceptible to proteolytic degradation [134]. The formation and accumulation of protein carbonyls (PC) has been one of the most commonly used methods for assessing overall protein oxidation in relation to exercise.

Increased protein oxidation evident by accumulation of O, O'-dityrosine [83] or PC have been reported by several authors [43,52,58,70,74,89,99,104,111], and have been shown to increase in a duration dependent fashion [43], as well as remain elevated for several hours (8 hours post) post aerobic exercise [52]. Null findings for PC post exercise are likely related to insufficient sampling times, training status of the subject population and/or short duration exercise protocols [47,64,66,69,82], as three of the five investigations noting no increase in PC utilized a GXT as the exercise stimulus, while only taking samples pre and immediately post exercise [47,64,66], while, subjects in the other two studies were considered to be well trained [69,82].

#### Antioxidant capacity

In response to conditions of strenuous physical work the body's antioxidant capacity may be temporarily decreased as its components are used to quench the harmful radicals produced. Thus measurement of the body's antioxidant capacity is utilized as a marker of oxidative stress. This is commonly assessed via the application of one of several antioxidant "capacity" assays (TEAC, FRAP, TRAP, ORAC) and/or the measurement of changes in specific antioxidant enzyme activity/concentration (SOD, GPx, CAT, GR).

It appears that the antioxidant capacity may be temporarily reduced during and immediately post exercise [50,94,95,115], after which time levels typically increase above basal conditions during the recovery period [50,52-54,58,87,91,111,115]. As with other markers, studies reporting no change in antioxidant capacity following exercise may have missed such changes by only taking one sample immediately post exercise [26,60,62,63], with the exception of one investigation which reported no change immediately post exercise, as well as 20 minutes post exercise [113].

Comparable to the antioxidant capacity response to exercise, specific enzymatic activity has been shown to respond in a similar manner. The antioxidant defense system may be reduced temporarily in response to increased RONS production, but may increase during the recovery period as a result of the initial prooxidant insult [50,115]. However, conflicting findings have been reported for each of the four main enzymes, with investigators noting increases in GPx [56,85,91,135], SOD [85,96,135], and CAT [52,54,58,85], as well as decreases in GPx [90], GR [135], SOD [95,136]. Furthermore, no change has also been reported for GPx [47,54,84,114,121,137], GR [84,137], SOD [47,56,78,105,137], CAT [47,56,84,137] activity following exercise. Clearly, these results are mixed and likely depend on the time of sampling, as well as the duration and intensity of exercise, which has varied considerably across studies.

#### Miscellaneous markers

In addition to those markers listed above, oxidative stress has also been assessed by way of a variety of other miscellaneous markers. These include circulating levels of individual antioxidants (e.g., vitamin E, vitamin C, beta-carotene), intermediates in purine metabolism (xanthine/hypoxanthine), as well as allantoin (product of the reaction between RONS and urate). Vitamin E is the major chain breaking antioxidant in vivo, as it serves to terminate the chain reaction of lipid peroxidation by reacting with the peroxyl radical [2]. Upon reaction with the peroxyl radical, vitamin E then becomes a radical itself, which is subsequently reduced by way of vitamin C (the major antioxidant in aqueous environments), forming yet another radical (vitamin C radical), which is further reduced by GSH [5]. Beta-carotene is a precursor to vitamin A in vivo, where it functions to suppress singlet oxygen [138].

In terms of circulating antioxidants, no change has commonly been observed [67,87,89,106,115,129,139], despite a few investigations reporting a transient decrease [75,89,139] or increase [77,106,115] immediately post exercise. Moreover, levels of reduced vitamin C have been found to decrease immediately post exercise [48,50], with one study noting a post exercise increase during the recovery period [50]. During conditions of oxidative stress, such as during exercise, increased circulation of antioxidants may result from an increased release from tissue pools, in turn sparing the quenching of other components of the antioxidant defense system [140]. In addition to the changes in antioxidants, other studies have reported an increase in xanthine /hypoxanthine following a variety of exercise protocols [76,122,128,129,141-143], with two studies also noting an increase in allantoin [122,144].

### Short to moderate duration protocols: impact of antioxidant supplementation

Of the above reviewed studies, several investigators have also included a variety of antioxidant treatments in their study design, in an effort to attenuate and/or eliminate exercise-induced oxidative damage. For a summary of such studies, please refer to Table 2 in Additional file 1. Typical treatments have included vitamin C, vitamin E, and beta-carotene, either alone or in combination for a variety of durations, administered chronically (1–8 weeks pre exercise) and acutely (1–2 days pre exercise). Vitamin E is believed to be the most important and effective nutritional antioxidant throughout the lipid phases of the cell, as it contributes to membrane stability and fluidity by preventing lipid peroxidation, whereas vitamin C plays an equally important role of preventing lipid peroxidation in plasma and interstitial fluids [140]. Moreover, vitamin C and vitamin E work in conjunction with each other during conditions of oxidative stress, as vitamin C is utilized to regenerate vitamin E following reaction with RONS [140]. Although not as commonly utilized, the major carotenoid precursor to vitamin A, beta-carotene, is primarily responsible for quenching singlet oxygen [140]. Aside from the common antioxidants above, other investigators have utilized less common antioxidants, including: coenzyme Q10 (CoQ10) [100], N-acetylcysteine (NAC) [63,120], uric acid [113], propranolol [101].

CoQ10, also known as ubiquinone, is an essential chemical component of the mitochondria in all animal cells where it functions as a cofactor in the electron transport chain during the synthesis of adenosine triphosphate (ATP) [145]. In addition to its role in energy production, CoQ10 has also been shown to provide antioxidant protection either by directly scavenging superoxide produced during oxidative phosphorylation or by regenerating vitamin C, and vitamin E from their oxidized states [146]. NAC is a thiol-containing compound which potentially may reduce the impact of RONS-associated damage by directly scavenging RONS and/or supplying cysteine for enhanced glutathione synthesis [120]. Uric acid is an abundant aqueous antioxidant that accounts for almost two thirds of all free-radical-scavenging activity in human serum [113]. Finally, propranolol is a β-blocking agent that has been shown to possess antioxidant properties in vitro [147].

Several studies have noted an attenuation in oxidative stress following administration of a variety of mixed antioxidant supplements (e.g., vitamin C and vitamin E/vitamin C, vitamin E and beta-carotene) [89,98,99,137]. However, independent or combined administration of vitamin C, vitamin E, and beta-carotene have been the most commonly utilized treatment option in regards to non-eccentric aerobic exercise-induced oxidative stress. Several studies have reported a reduction in exercise-induced oxidative stress following chronic administration of vitamin C [62,70,119], vitamin E [21,55,80,139], and beta carotene [129] when administered alone. However, attenuation has not occurred for all measured biomarkers [62,70,80,139]. Moreover, a few studies have reported no effect of independently administered vitamin C [98], or vitamin E [66,90,98]. Null findings were also reported following independent administration of CoQ10 [100]. Disparities in the literature regarding antioxidant supplementation and attenuation of oxidative damage are likely due to several factors including training status of the subject population [135], dietary intake [115], as well as the magnitude and duration of supplementation period, as both vitamin C [70] and vitamin E [148] have been shown to respond in a dose-dependent manner. The null findings in regards to vitamin E supplementation [66,90,98] could have been due to insufficient dosages and or treatment durations, as it has recently been shown in a time-course study, that maximal reduction of oxidative stress (assessed via F_2_-isoprotanes) does not occur until 16 weeks of vitamin E supplementation at a dosage of at least 1600 IU per day [148]. It is certainly possible, though not reported to date, that other antioxidants respond in a similar manner and could potentially explain a portion of the inconsistency regarding antioxidant supplementation in attenuating exercise-induced oxidative stress.

Acute antioxidant supplementation prior to or during non-eccentric aerobic exercise, although not as commonly investigated, has resulted in more consistent findings when compared to chronic supplementation. This is evidenced by an attenuation in various biomarkers of oxidative stress following treatment, almost without exception [27,62,63,69,80,101,113,120]. Attenuated biomarkers have included PC [69], 8-OHdG [69], DNA damage via Comet Assay [80], GSSG [63,120], TBARS [62], MDA [27], LOOH [27], F_2_-isoprostanes [113], total antioxidant capacity [63,113], and expired pentane [101]. These results have been noted following acute administration of a multivitamin [80], vitamin C [27,62], vitamin E [80], NAC [63,120], uric acid [113], propranolol [101], as well as an antioxidant (black grape, raspberry, red currant concentrates) rich beverage [69]. Furthermore, direct detection of exercise-induced RONS production, via electron spin resonance, has also been shown to be eliminated following acute ingestion of 1000 mg of vitamin C [27]. It should be noted, that in a similar manner to chronic supplementation, no antioxidant treatment completely eliminated oxidative stress, as attenuation was not consistent across all selected biomarkers for any study [62,63,69,80,101,113,120], with one exception [27].

### Eccentric bias

While most investigations have implemented primarily concentric aerobic regimens (e.g., cycling, treadmill walking/running), some have measured the oxidative stress response following aerobic exercise with an eccentric bias, as discussed below. For review, please consult Table 3 in Additional file 1.

Eccentric exercise involves high force during the lengthening portion of muscle contraction. This can occur involuntarily or voluntarily during conditions in which the activated muscle cannot produce enough force to overcome the resistive force (e.g., during heavy resistance training) or during an intentional production of submaximal force in order to control the eccentric (lengthening) movement (e.g., controlled lowering of external load and/or downhill running), respectively. Both damage to the involved muscle tissue and concomitant soreness associated with such damage have been shown to be greatest following eccentric compared to concentric exercise [149]. Furthermore, exercise-induced trauma to the musculature has been shown to lead to a proinflammatory migration of phagocytic cells into the affected area, leading to the increased release of RONS (during respiratory burst), designed to aid in the breakdown of damaged tissue [101,150-152]. Both the post exercise phagocytic migration, as well as the initial increase in RONS during eccentric exercise (due to increased mitochondrial respiration) have been suggested to result in an acute state of oxidative stress during and following an eccentric exercise stimulus [151,153,154].

Almost without exception, the majority of studies have utilized eccentric protocols in the form of downhill treadmill running. Typical intensities and durations have included running speeds corresponding to 70–75% age predicted heart rate max or 60% VO_2max _with a duration of 40–50 minutes of continuous or intermittent (3 bouts of 15 min downhill runs) exercise at a negative 12–20% grade. This form of exercise is often chosen in order to induce muscle tissue damage, evident by reported increases in creatine kinase (CK) [153-156] and lactate dehydrogenase (LDH) [156] following such a protocol. Along with these markers of cellular damage, studies have reported mixed finding in relation to oxidative stress biomarkers.

Increased lipid peroxidation, measured via TBARS [156], MDA [154,157,158], F_2_-isoprotanes [45,154], and LOOH [155], has been reported by several authors following aerobic eccentric protocols in untrained subjects, with elevations typically reaching significance several hours (> 6 h) or days (24–72 h) following the stimulus. This would provide evidence for the increased migration of phagocytic cells following eccentric exercise, resulting in increased RONS production and subsequent oxidative damage. In opposition to the above findings, two similar investigations, utilizing trained subjects noted no changes in MDA [153], conjugated dienes [153,155], or glutathione redox status [151]. It was suggested that trained individuals may experience an attenuated oxidative stress response following eccentric exercise, perhaps mediated by greater antioxidant enzyme protection and/or lower levels of muscular damage following exercise [153]. Furthermore, the null findings of Camus et al. [151] may have been related to sampling time, rather than training status, as samples were only taken immediately and 20 minutes post exercise.

Aside from lipid peroxidation, other biomarkers have been utilized by a few investigators, including markers of DNA damage (8-OHdG), as well as changes in antioxidant capacity (ORAC) and/or circulating levels of antioxidants (vitamin C, vitamin E). Only one study to our knowledge has investigated oxidative damage to DNA, as well as changes in antioxidant capacity following eccentric exercise, noting no increase in 8-OHdG and a decrease in ORAC evident at 72 h post protocol [154]. In regards to circulating antioxidants, blood levels of vitamin C and vitamin E have been shown to exhibit no change when corrected for changes in plasma volume [153,154,158]. Although, other work opposes these findings, noting a transient decrease in the plasma concentration of vitamin C [151] and vitamin E in skeletal muscle [158].

As with non-eccentric biased aerobic exercise, a few investigations have included antioxidants within the research design involving eccentric aerobic work. Due to the relatively small number of such studies, these can also be reviewed in Table 3 of Additional file 1. Vitamin E, provided at a dosage of 1000 and 1600 IU per day for 12 weeks or 48 weeks was reported to attenuate the increase in F_2_-isoprostanes following eccentric exercise [154], as well as eliminate the increase in urinary MDA which was observed 12 days post exercise in the placebo group [158], respectively. In support of Meydani et al. [158] a similar study, utilizing vitamin C (dosage of 1 g/day), noted no increase in MDA following eccentric exercise, compared to a significant increase 3 and 4 days post exercise in the placebo group [157]. It should be noted however that the treatment effect observed by Sacheck and coworkers [154] was only evident in older subjects, with young healthy subjects experiencing no additional benefit of supplementation. In such a case, it may be that antioxidant supplementation only provides additional protection in those individuals at an increased risk of oxidative damage due to the presence of old age and/or disease [159]. Moreover, a reduction in exercise-induced oxidative stress by way of antioxidant supplementation may not be beneficial, as it has been suggested that increased RONS during and following exercise may be necessary in order to bring about adaptations in antioxidant defenses, as well as other physiological parameters [19,31,36].

### Long duration protocols

The idea of exercise-induced oxidative stress representing a potential contributor to the development and/or progression of ill health and disease receives considerable attention when applied to acute long duration (> 2 hours) aerobic exercise, (for review see [140]). In fact, epidemiological data suggests that a very high volume of exercise is associated with an increase in the risk of developing cardiovascular disease [160,161]. Moreover, increased oxidative stress has been suggested to be the link, connecting the above association between excessive exercise and disease risk [140]. At first glance, this statement appears to be highly contradictory to common beliefs regarding regular exercise and health benefits, as current recommendations suggest that individuals should accumulate at least 30 minutes of moderate-intensity physical activity each day in order to improve and maintain their health [162]. These recommendations are made in spite of the fact that numerous studies have reported increased oxidative stress in response to acute aerobic exercise of various intensities and durations (for review see relevant section above). Collectively, disease risk has been shown to decrease as a function of exercise up to a certain point, at which the disease risk begins to increase, suggesting that an optimal level of exercise may exist [140]. Because oxidative stress appears connected to the relationship between disease and exercise, it is certainly possible that an optimal level of increased RONS production during exercise gives to way to improved health, potentially via an upregulation in antioxidant defenses. However, because RONS production is known to be a function of both exercise intensity [41] and duration [43], exacerbated prooxidant production that exceeds the currently undefined optimal level, may in turn overwhelm antioxidant defenses in such a way that irreparable oxidative damage may occur, potentially resulting in ill health and or disease. More research is needed before definitive conclusions can be established, however, several studies have investigating the oxidative stress response following long duration aerobic exercise. For the purpose of this review, long duration aerobic exercise will be defined as aerobic activity maintained for a duration of greater than two hours and/or performed in a field setting (e.g., half or full marathon). Additionally, the impact of acute overtraining on oxidative stress will also be included in this section. These studies will be reviewed in detail below and will be presented in Tables 4 (without antioxidant supplementation) and 5 (with antioxidant supplementation) in Additional file 1.

Long duration exercise-induced oxidative stress has typically been assessed following either a half [163-167] or full [131,168-176] marathon, an ultramarathon [177-182], or a triathlon [183-188]. Although other findings have been reported in reference to a duathlon [189-192], a long duration run [71,169,193-197], cycle ride [198,199], march [200], or bike race [71,201-204]. Studies investigating the impact of overtraining have also been conducted [191,192,205,206]. Collectively, it would appear that acute long duration aerobic exercise promotes an acute state of oxidative stress, evident by reported increases in lipid peroxidation (TBARS [190,191], MDA [44,163,164,166,168,203,204], F_2_-isoprostanes [177,178,180,181,187,188,194,197,199,207] CD [71,169,193] LOOH [177,178,197], susceptibility of LDL to oxidation [170,172,175,193]), protein oxidation (PC) [203], oxidative damage to DNA (8-OHdG [168,182,201], DNA damage (Comet assay) [168,174,200,208]), as well as changes in GSH redox status (decreased GSH [165,173,190-192,195] and increased GSSG [165,173,190-192,195,202,203]). However, a few exceptions have been noted such as no change in TBARS [71,165,171,176,183,192,195], MDA [164,196], F_2_-isoprostanes [179], CD [165,166,170], LOOH [184,187,188], susceptibility of LDL to oxidation [167], PC [200], 8-OHdG [185,188], and glutathione redox status [183]. Exercise-induced changes in antioxidant defenses follow a similar pattern as the results presented in the previous section on non-eccentric aerobic exercise, with antioxidant capacity typically experiencing an increase immediately post race [163,164,169,170,172,175,187,192,197,199]. Varying results for specific antioxidant enzymes, as well as circulating antioxidants have also been reported by several authors, noting a transient increase (GPX [176,189,196], GR [189,202,203], SOD [166,203], CAT [202], vitamin A [165], vitamin C [165,172,175,207], vitamin E [169,181,202]), decrease (GPX [175,176,181,207], SOD [44], CAT [44,166], vitamin A [184], vitamin C [170,176,181], vitamin E [175,176,181,207]), or no change (GPX [165,166], SOD [165,173,189,192,202], CAT [165,189,203], vitamin A [170,176,202,203], vitamin C [170,184], vitamin E [165,170,172,184,203]) following exercise. Null findings for any of the above biomarkers have been suggested to be related to the highly trained nature of the subject populations, intensity of exercise, biomarkers utilized, the timing of tissue sampling [140], as well as the uncontrolled intake of carbohydrates [140] and nonsteroidal anti-inflammatory drugs (NSAID) [179]. On average, subjects participating in the above investigations trained approximately 20–30 hours/per week and thus likely experienced decreased RONS production, as well as increased antioxidant defenses [140,183]. It was suggested that while the duration of some exercise protocols may have been sufficient for the induction of RONS production, the intensity was likely so low (in order to maintain the long duration activity), that such highly trained individuals may have possessed sufficient antioxidant defenses to combat such radical production, thus masking any potential accumulation of oxidative stress biomarkers [183]. Similar to aerobic eccentric exercise, long duration protocols are known to result in substantial muscle damage (evident by increased CK [165,168,182,200,201]), subsequently resulting in phagocytic migration to the affected area, increased respiratory burst activity and oxidative stress. Therefore, if sampling was not carried well into the recovery period, oxidative stress may not have been identified. Moreover, the lack of sampling during the actual protocol itself may also have impeded investigators ability to detect an oxidative stress, as elevations have been reported during such protocols [163,181]. Finally, as mentioned above, lack of control for both carbohydrate and NSAID intake during exercise may also have influenced results as both have been shown to attenuate [199] and exacerbate [179] oxidative stress, respectively.

A few studies have investigated the impact of overtraining for a period of days or weeks on various markers of oxidative stress. Overtraining protocols have included some form of vigorous exercise, performed for a defined length of time, such as 10 [205], 28 [192], or 30 [206] days, typically reporting an increase in oxidative stress following cessation of training (8-OHdG [205,206], TBARS [206]). However, in opposition to the above findings, one study reported no increase in markers of lipid peroxidation, DNA damage or glutathione redox status following a period of overtraining in trained men [192].

### Long Duration Protocols: Impact of Antioxidant Supplementation

Numerous studies have investigated the impact of antioxidant supplementation on long duration exercise-induced oxidative stress. These studies are presented in Table 5 of Additional file 1. Treatments have typically consisted of the common antioxidants (vitamin A, vitamin C, vitamin E) administered in combination [174,176,190,191,196,198,207] or separately [177,180,187-189,209], with the exception of a few studies utilizing CoQ10 [175], as well as acute administration of carbohydrate-rich beverages, with [178] or without [197,199] additional vitamin C.

Unlike the results of antioxidant treatment and short duration aerobic exercise discussed above, the majority of investigators have noted no attenuating effect of supplementation on markers of lipid peroxidation, DNA damage, and/or glutathione redox status following long duration protocols [175,177,191,197]. However, some exceptions exist, with authors reporting reductions in F_2_-isoprostanes [180,199,207], TBARS [198,209], as well as DNA damage (Comet assay) [174] following supplementation with vitamin C and vitamin E administered in combination ([174,198,207], as well as vitamin C [199] and vitamin E [180,209] given separately. While the lack of enhanced protection against oxidative stress may be related to the issues discussed above (e.g., training status, dosages, time course of supplementation), it may be that the increase in RONS observed during and following long duration protocols may be so great that the prooxidants produced overwhelm both the endogenous and exogenously consumed antioxidant defenses, thereby masking the benefit of supplementation. It is possible that larger dosages and or longer durations of treatment may be necessary in order to provide significant protection against long duration exercise-induced oxidative stress [148].

### Aerobic exercise and oxidative stress: summary

It has been shown that exercise of various intensities and durations serves as a sufficient stimulus to invoke increased RONS production in both animals [25] and humans [92]. While the body does possess a complex antioxidant defense system that serves to provide protection against RONS, defenses are often not sufficient to eliminate oxidative damage during and following exercise, evident by numerous findings of increased lipid, protein, DNA and glutathione oxidation following acute aerobic exercise (both short and long duration protocols) in humans and animals. Antioxidant supplementation does appear to provide some degree of protection, typically observed with short duration protocols; however, precise dosages and durations of treatment remain to be determined. Both the oxidative stress experienced following exercise, as well as the impact of antioxidant supplementation appears affected by several factors including intensity and duration of exercise, training status, age, and health status of the subjects tested, in addition to the specific biomarkers chosen, timing of tissue sampling, and the amount and duration of antioxidant treatment. Therefore, it is recommended that future investigations employ sufficiently stringent exercise protocols, and utilize a wide array of oxidative stress biomarkers and take multiple samples post exercise (through several hours of days of recovery) in an attempt to provide valid and meaningful findings.

Although much has been uncovered regarding oxidative stress and exercise, it is currently unclear as to whether exercise-induced RONS production and subsequent oxidative damage represents a necessary or detrimental stimuli to physiological function that should be utilized or minimized, respectively. It may be that a currently undefined optimal level of RONS production and oxidative damage is necessary for adaptations in antioxidant defenses and other physiological parameters that lead to the improvement of proper health. If so, this may provide insight into the relationship between regular physical activity, diminished disease risk, and increased life expectancy [160,161,210]. However, excessive RONS production and oxidative damage via chronic long duration exercise and/or overtraining may exceed the aforementioned optimal level, thereby leading to irreparable oxidative damage, potentially resulting in the development or progression of ill health and/or disease. If such was the case, this finding may provide insight into the relationship between excessive exercise, increased disease risk, and decreased life expectancy [160,161,210]. Clearly, more research is needed in this area in order to generate firm answers related to these issues.

### Acute Anaerobic Exercise: Human Studies

Although the term anaerobic means "without oxygen", resistance training does result in increased oxygen consumption both during and following acute exercise. However, the magnitude of increase in VO_2 _is far less than what is observed following acute aerobic exercise [211]. Despite the comparatively low increase in VO_2_, it has been shown that acute anaerobic exercise serves as a sufficient stimulus to elicit an increase in RONS formation [28,29]. Furthermore, unlike aerobic exercise, where increased mitochondrial respiration is thought to be the primary target of increased RONS, it has been suggested that the increased radical production and subsequent oxidative stress observed during and following resistance exercise may be meditated to a large degree by the activities of certain radical generating enzymes (xanthine and NADPH oxidase), prostanoid metabolism, phagocytic respiratory burst, disruption of iron containing proteins, as well as altered calcium homeostasis [24]. Brief periods of ischemia followed by reperfusion, resulting from intense muscular contraction, as well as mechanical stress and/or muscle damage, are thought to be the mechanisms underlying the increase in RONS via triggering the activity of radical generating enzymes as well as initiating the migration of inflammatory cells to the affected area [20]. Similar to aerobic exercise, although the mechanisms are not fully understood, anaerobic exercise clearly possesses the ability to result in acute oxidative stress, evident by several studies reporting an increase in oxidative stress biomarkers following exercise [24]. For this review, results will be discussed relative to the mode of resistance exercise (e.g., dynamic, eccentric, isometric, sprint/jump), and will be presented accordingly in Tables 6–9 of Additional file 1. Because of the relative infrequency of such studies, those incorporating antioxidant treatment into their design will not be discussed in a separate section, but rather they will be included within their respective section and table.

### Dynamic resistance exercise

The majority of studies investigating dynamic resistance exercise-induced oxidative stress (Table 6 of Additional file 1) have utilized an exercise protocol consisting of two or more compound lifts (multiple joint exercises), occasionally performed in a circuit fashion [212-214], for ≥ 3 sets at an intensity of 60–95% 1 RM [212-221]. Other studies have used a single movement, such as the squat [222-227] or knee extension [28,29] exercise as the stimulus, with the exception of one study in which isokinetic knee extension was performed following maximal sprints on a cycle ergometer [228].

Similar to aerobic exercise, the majority of studies have reported an increase in oxidative stress, evident by increased lipid peroxidation [28,29,212,214-216,218-221,223,225], protein oxidation [216,224,229], and changes in glutathione redox status [217,224,226], despite a few studies noting null findings for each (lipid [212,213,222,224,226-228], protein [226,227], glutathione [218,219]). In regards to DNA oxidation, no study has reported significant increases following dynamic resistance exercise [222,224]. Assessment of antioxidant capacity, concentrations of circulating antioxidants, as well as the activities of certain antioxidant enzymes has resulted in similar inconsistent results to those observed with aerobic exercise, with authors reporting an increase, decrease or no change for various markers (for more information, consult Table 6 of Additional file 1). Null findings are likely related to the specific biomarkers chosen, time course of sample collection, intensity of exercise [221], dietary intake, as well as the training status of the subject population [212,213,222,224,227]. As with aerobic exercise, it may be that oxidative stress occurred but it did so preceeding or following the sample collection, in a different tissue other than that utilized (typically blood and urine), or resulted in oxidative damage to cellular constituents other than those measured. Furthermore, trained individuals likely experience attenuated muscular damage in response to exercise compared to untrained subjects, in turn blunting the inflammatory and subsequent oxidative stress response.

In an attempt to decrease the oxidative damage induced by exercise, a few studies have investigated the impact of various antioxidant supplements and/or agents [213-215,217,220,225,228]. Attenuation of exercise-induced oxidative stress has been reported following administration of exogenous vitamin E [214,228], L-carnitine [225], and allopurinol [217], despite no treatment effect being noted following similar vitamin E intake [215,216], as well as following acute ingestion of a carbohydrate beverage preceeding and during exercise [213].

### Eccentric biased resistance exercise

In the assessment of eccentric biased exercise-induced oxidative stress the majority of protocols involve eccentric contractions of either the elbow flexor [229-235] or knee extensor [229,231,236-238] muscles. The exceptions include those studies in which eccentric exercise was performed on a cycle ergometer [239] or using eccentric bench press [240]. These studies can be viewed in Table 7 of Additional file 1. Such protocols have been suggested to result in increased muscle damage/cell membrane disruption, evident by increased CK following exercise [229,231,233,238-241]. Furthermore, in an effort to produce the greatest amount of trauma to the exercising muscle, the majority of studies have recruited untrained subjects [229,230,233,239], with few exceptions [238,240].

Such protocols have been shown to result in increased lipid peroxidation [230,231,237,238], protein [230,233,237,238] and DNA [236] oxidation, as well as changes in glutathione redox status [230,234,235,237,238]. Moreover, values have been shown to peak 48–72 hours post exercise, suggesting that increased migration of phagocytic cells and subsequent increased RONS production via respiratory burst may be the main determinant of the oxidative stress response [230,231,233,237,238]. However, null findings have also been reported despite similar exercise regimens for markers of lipid peroxidation [229,232,239-241], protein oxidation [229], and glutathione redox status [233]. These findings are likely related to the limitations discussed previously. A lack of significance may also be the result of an inability to induce muscular damage (evident by no increase in CK following exercise [232]), or the use of skeletal muscle, rather than blood, to measure oxidative stress [229,241]. Aside from the biomarkers discussed above, various antioxidant capacity assays, as well as the activity of specific antioxidant enzymes (e.g., SOD, GPx, CAT) have been shown to increase following exercise [231,237,238,241], with few exceptions [231,239].

Little information exists concerning eccentric exercise and antioxidant supplementation, however a few studies have noted an attenuation in oxidative stress following administration of vitamin C, vitamin E, and selenium given in combination [230], or vitamin C alone [235]. No benefit has also been reported following consumption of a vitamin E, omega-3 free fatty acids or soy isolate mixture [232], a vitamin C and vitamin E mixture [240] as well as following intake of vitamin C and NAC [231]. Moreover, the vitamin C, NAC combination was administered following exercise and into the recovery period and was shown to result in an exacerbated increase in oxidative stress compared to placebo [231].

### Isometric exercise

Isometric protocols have typically consisted of handgrip exercises with [242,243] or without [81,244-247] thumb adduction at 50–100% of maximal voluntary contraction (MVC) either until exhaustion [242,243,245,246] or for a specified amount of time [81,244,246,247]. Other studies have also utilized static knee extension at an intensity of 30 [248] or 66% MVC [249]. While prolonged isometric exercise is characterized by acute ischemic conditions, one study attempted to exacerbate the ischemic period by placing a blood pressure cuff (inflated to 30 mmhg above known systolic pressure) on the exercising arm during the protocol [247]. It is believed that the acute ischemia and rapid reperfusion observed during and following prolonged isometric exercise gives rise to increased RONS formation, perhaps via the radical generating enzyme xanthine oxidase [24]. Studies utilizing isometric protocols can be viewed in Table 8 of Additional file 1.

Though data are limited, the majority of the above studies have noted an increase in lipid peroxidation following exercise [81,242-245,247], as well as changes in the glutathione redox status [242,244,246,248] and decreased antioxidant capacity [244,245]. However, changes appear to be transient, rapidly returning to pre exercise levels within minutes following exercise [242,247]. The highly transient nature of changes in biomarkers may potentially, along with the previously discussed factors, explain some of the null findings [81,242,248,249]. Only one study to our knowledge has investigated the impact of antioxidant treatment, reporting an attenuation of glutathione oxidation following handgrip exercise when subjects were given an infusion of 100 ml of NAC during exercise [246].

### Sprint/jump exercise

The majority of studies investigating oxidative stress subsequent to sprinting exercise have utilized some form of fatiguing maximal effort sprint either on a cycle ergometer [30,250-254] or running surface [255,256]. Additionally, studies incorporating both an intermittent shuttle run [257-259], as well as a 100 m and 800 m swim [260] will also be discussed in this section. In regards to jumping exercise, one study measured oxidative stress in response to six, 30 second sets of repeated jumping in trained and untrained men [261]. These investigations are presented in Table 9 of Additional file 1.

Results for the sprinting studies are much more contradictory than those of the previous section, with a similar number of studies noting both an increase in lipid peroxidation [30,251,256], protein oxidation [252], and DNA damage [255], as well as no change in lipid [250,252-254], protein [250], and DNA [252] oxidation. It may be that the volume of exercise, and/or the resistance applied during sprinting was insufficient to evoke an oxidant stress, as lipid peroxidation has been shown to increase as a function of the resistance applied to the flywheel during cycle sprinting [251]. Moreover, a longer duration intermittent shuttle run has been shown to result in increased lipid peroxidation, assessed via increased concentrations of MDA [257-259], with both a null and significant attenuating effect offered by acute [257,258] and chronic [259] administration of vitamin C prior to the run, respectively. Null findings have also been reported following supplementation for 20 days with Coenzyme Q10 prior to an intermittent maximal sprint test on a cycle ergometer [254].

In regards to other forms of high intensity anaerobic exercise, both successive jumping exercise [261], as well as intense swimming [260] resulted in no change in lipid peroxidation and a decrease in reduced glutathione, respectively.

### Anaerobic exercise and oxidative stress: summary

It has been shown that anaerobic exercise results in increased RONS production and collectively, it appears that all forms of anaerobic exercise possess the ability to result in increased oxidative stress. The mechanisms responsible for the exercise-induced increases in RONS have been suggested to be largely a function of radical generating enzymes (activated in response to ischemia followed by reperfusion) and/or phagocytic immune response following muscle damaging exercise. Similar to aerobic exercise, a variety of factor likely impact the oxidative stress response observed, including, specific biomarkers chosen, time course of sampling, tissues sampled, intensity and volume of exercise, as well as the training status and dietary intake of the subjects. The use of antioxidant supplements has given rise to conflicting results with some studies noting an impact, despite other similar studies reporting no additional benefit of supplementation. Taken together, the results of the anaerobic research are not unlike those of aerobic nature; there are simply fewer data on the former compared to the latter. As with aerobic exercise, it is currently unclear as to whether increased RONS formation observed during anaerobic exercise represents a necessary or detrimental event.

### Sporting events

Sporting events often possess components of both an aerobic and anaerobic nature and are typically performed in an outdoor, uncontrolled setting. Thus, such studies are discussed in a separate section and are presented in Table 10 of Additional file 1. A few investigators have examined the oxidative stress experienced following sporting events including football [262], basketball [263], soccer [264,265], rugby [266,267], motocross racing [268], and professional climbing [269]. While most did in fact measure oxidative stress following an acute session [262,265,266,268,269], others simply assessed changes in biomarkers at rest following a prolonged period of regular season training [263,264,267].

Related to football, one study noted an increase in lipid peroxidation (measured via increased total peroxides and antibodies against oxLDL) following a professional American football game [262]. Similar increases in lipid peroxidation have also been noted following a rugby match [266] and soccer practice [265], with untrained rugby players experiencing exacerbated increases in lipid peroxidation compared to their trained counterparts [266]. Moreover, trained athletes have been shown to possess higher levels of antioxidant protection [267], as well as lower levels of resting lipid peroxidation [264] compared to sedentary controls. Both continuous climbing to exhaustion, as well as a simulated motocross race resulted in an increase in MDA, PC, GSSG, and TAC [268,269], with climbing exercise also inducing a decrease in GSH and TGSH [269].

Although various sporting events appear to result in increased oxidative stress, it is likely that the vigorous training accompanied by such events leads to an up-regulation in antioxidant defenses, thereby protecting individuals from excessive oxidative damage. However, as may be the case with long duration aerobic exercise, athletes participating in a high volume of vigorous exercise may benefit from antioxidant treatment, as supplementation has been shown to result in decreased oxidative stress and increased antioxidant defenses in professional basketball players [263].

### Acute aerobic and anaerobic exercise: animal studies

The data presented thus far has been relative to investigations using human subjects. However, an extensive body of research is available with regards to exercise-induced oxidative stress in animal models. Because of the volume of this work, in addition to the fact that multiple tissues and biomarkers are often studied, each individual investigation will not be presented in table format. Rather, a brief synopsis of this work will be presented below.

First and foremost, it should be noted that the results of research utilizing animal models are not unlike those using human subjects in that most demonstrate an increase in oxidative stress biomarkers with acute exercise. It should also be noted that there exists more consistency in the reported findings with the animal work, likely due to the homogeneity of animals and the great degree of control that can be implemented in these designs. The vast majority of investigators have reported increases in various oxidative stress biomarkers in several tissues following a myriad of both aerobic [25,270-292] and anaerobic [293-298] exercise protocols. Null findings for lipid [272,298-302], protein [279,300,303], and glutathione [290,304] oxidation are far more scarce than those seen in human studies, which could potentially be explained by the much more controlled nature of animal research as well as the feasibility of measuring a variety of oxidative stress biomarkers in several biological tissues (e.g., heart, brain, lung, kidney, diaphragm, skeletal muscle, blood).

### Acute exercise and oxidative stress: effect of gender

In a study conducted by Ruiz-Larrea et al. [305], the female sex hormone estrogen was shown to exhibit antioxidant properties in vitro, and because females possess a larger concentration of estrogen compared to males, it was believed that they may be less susceptible to oxidative stress [172,186]. Evidence in support of this notion has been provided by both animal and human studies, although gender differences appear much more pronounced when utilizing animal models, as female rats run to exhaustion have shown modest if any exercise-induced oxidative stress [306] as compared to male rats [307]. In addition to an attenuated response following acute exercise, female rats have also been shown to possess lower resting levels of oxidative stress compared to males [308]. However, estrogen may not be the only factor involved in gender comparisons of oxidative stress [306], as vitamin C, vitamin E and glutathione levels were also reported to differ in male and female rats following an acute exercise bout [309] as well as at rest [308]. Moreover, estrogen administration to male rats resulted in a decrease in vitamin C levels within the muscle [310], providing evidence that alternative mechanisms other than increased estrogen may play a role in explaining the attenuated oxidative stress response observed in the above investigations.

In regard to studies conducted utilizing human subjects, Chung et al. [86] investigated the role of estrogen in decreasing exercise-induced oxidative stress and found minimal difference in oxidative stress levels of women during both the luteal and follicular phases of their menstrual cycle. In support of Chung and coworkers [86], several other studies have reported no difference in the exercise-induced oxidative stress response between men and women following both submaximal aerobic [43,99,114], long duration aerobic [172], and isometric [246] exercise. It should be noted that although no differences were reported following acute exercise, women have been shown to possess decreased oxidative stress, as well as increased antioxidant protection at rest compared to men [99,114,311]. In opposition to the above findings, Ginsburg et al. [186] reported a decrease in the susceptibility of plasma lipids to peroxidation in men following a triathlon, with no significant change being noted in women. However, uncontrolled antioxidant supplementation occurred in the study and women were 10 yrs older than men and their activity time was about 150 minutes longer with exercise intensity not matched [186].

Collectively, it appears that both men and women are susceptible to oxidative stress at rest and during exercise. Female resting levels of oxidative stress markers may be lower, but exercise-induced oxidative stress responses appear similar between genders. Women's lower resting levels could in part be due to their higher expression and activity of antioxidant enzymes and could potentially explain their longer life span [308].

### Oxidative stress and chronic exercise: role of hormesis

Clearly, acute exercise imposes a physical stress on the body, as numerous studies have shown that oxidative stress biomarkers are increased following both aerobic and anaerobic exercise. However, whether this exercise-induced increase in RONS exerts detrimental effects on long term physiological function remains a topic of debate, as an ever increasing body of evidence in the area suggests that biologically-derived RONS act in a hormetic manner [9,312,313]. That is, in response to repeated exposure to toxins and/or stressors the body undergoes favorable adaptations that in turn result in enhanced physiological performance and improved physical health [9,313]. Thus, an optimal level of RONS production appears conducive to optimal health, whereas too little or too much RONS result in impaired defense capabilities or extensive oxidative damage and inflammation, respectively, both of which would be expected to promote the development of ill-health and/or disease. The above concept is perhaps best exemplified when applied to the effects of exercise-induced RONS production on the intracellular redox balance. Recall from above that the redox state present within individual cells has been suggested as a key component of gene expression, as well as cell function, and that chronic disregulation of such balance in favor of a more oxidizing environment is associated with the development of numerous diseased states, in addition to the aging process [16]. Moreover, because a more reducing environment is believed to promote health-enhancing effects [16], interventions designed to shift the redox balance in favor of greater reducing potential via increasing antioxidant defenses appears warranted.

One such method that appears to exert powerful benefits in terms of increasing antioxidant protection is the performance of regular moderate intensity exercise [9]. This upregulation in antioxidant defense observed with regular exercise training would be expected to shift the redox balance in favor of more reducing conditions, thereby potentially explaining the pro-health/anti-pathological effects of exercise [16,313]. The mechanism whereby regular exercise results in an adaptive benefit is well described [9]. In brief, exercise-induced RONS appear to serve as the "signal" needed for the activation of MAPKs (p38 and ERK1/ERK2), which in turn activate the redox sensitive transcription factor NF-κB [36], via activation of IκB kinase, which then phosphorylates IκB (the inhibitoy subunit of NF-κB). IκB is then ubiquinated and subsequently degraded via the cytosolic ubiquitin-proteosome pathway, thereby releasing NF-κB to migrate into the nucleus. Several antioxidant enzymes [manganese superoxide dismutase (MnSOD), inducible nitric oxide synthase (iNOS), glumatylcysteine synthetase (GCS)] contain NF-κB binding sites in their gene promoter region and thus are potential targets for exercise-induced upregulation via the NF-κB signaling pathway [9]. Therefore, any attempt to attenuate the exercise-induced increase in RONS production (via antioxidant supplementation) may actually blunt the adaptive increase in antioxidant defenses and subsequent desirable shift in redox balance, thereby increasing an individual's susceptibility to disease and prooxidant attack both at rest, as well as during subsequent exercise bouts [36,44].

Evidence in support of this notion is provided by the reportedly blunted exercise-induced upregulation in MnSOD, iNOS, reduction in phosphorylation of p38 and ERK1/ERK2, as well as reduced activation of NF-κB in response to allopurinol (a known inhibitor of xanthine oxidase) administration [36]. Additionally, in both human and animal models, supplementation with vitamin C has been shown to blunt adaptive increases in VO_2max_, as well running to exhaustion [312]. Similar results in terms of reduced exercise performance following antioxidant supplementation have been reported with the use of vitamin C [314], vitamin E [315] and ubiquinone-10 [316] in greyhounds and humans, respectively. It should be understood that the potential negative effects of antioxidant supplementation may exist only when applied to moderate intensity exercise, as administration of antioxidants during competitive and/or exhaustive exercise training periods has been shown to attenuate markers of muscle damage and lipid peroxidation [317].

Collectively, it would seem that an optimal level of RONS produced during exercise is not only necessary, but advantageous in that it serves to drive the desired adaptive response. In support of this notion, adaptations that occur to the body's antioxidant defense system in response to regular exercise appear to not totally eliminate oxidative damage, but merely reduce potential damage from future acute bouts of exercise [24,318], as well as other ROS generating situations. These findings support the idea that complete elimination of exercise-induced RONS would not be conducive to optimal physiological function. On the contrary, the production of RONS above and beyond that currently undefined level, potentially as a consequence to conditions similar to overtraining (chronic performance of vigorous exercise), may serve to overwhelm the defense system in place, thereby resulting in extensive oxidative damage, decreased performance and ill-health/disease, as evidenced by the increase in disease risk associated with ultra-endurance exercise training [44]. At present, it would seem prudent for future research within the area of oxidative stress and exercise to focus attention towards further elucidating this critical limit between desirable and detrimental effects of exercise-induced RONS. This information is important in informing athletes and coaches, exercise enthusiasts and trainers, clinical populations and practitioners, as well as the general population as to the need for antioxidant supplementation within the context of regular exercise. This work may also provide information as to the volume of exercise conducive to beneficial health outcomes.

## Conclusion

At present, it appears that all forms of exercise, both aerobic and anaerobic, possess the potential to result in increased RONS production and subsequent oxidative stress in both human and animal models. It should be understood that results presented above are in relation to otherwise healthy individuals. A handful of investigations have been conducted addressing exercise-induced oxidative stress in diseased populations including cardiovascular disease [77,78], intermittent claudication [65], diabetes [61,79,319], hypercholesterolemia [96], obesity [60,110], and chronic obstructive pulmonary disease [320], as well as in cigarette smokers [74,321]. These investigations have typically noted an exacerbation in oxidative stress in diseased subjects compared to healthy controls [60,61,65,74,78,110,319,321]. Aside from disease status, several other factors appear to play a significant role in the exercise-induced oxidative stress response including mode, duration, and intensity of exercise, specific biomarkers chosen, time course of tissue sampling, as well as the training status and dietary intake of the subject population. Discrepancies in the literature are likely related to the above factors, as well as individual differences inherent with human research.

In the past, the relationship between exercise and oxidative stress has commonly been viewed as a detrimental phenomenon that should be reduced or eliminated in an effort to improve performance and/or health, with studies reporting conflicting results following antioxidant supplementation. While excessive RONS production and oxidative stress certainly has the ability to result in physiological damage, perhaps leading to the development of ill-health and/or disease over time, an optimal level of prooxidant production may actually serve as the necessary stimulus for the upregulation of antioxidant defenses, thereby providing protection against future RONS attack and disease development. Although the role of oxidative stress in exercise-induced adaptations, as well as in human physiology remains to be completely elucidated, it appears based on the extensive body of literature that a currently undefined optimal level of RONS production may be imperative in order for optimal adaptive potential and physiological function to be achieved. It may no longer be prudent to view prooxidants produced during exercise as harmful agents, but rather as a useful mechanism that can be manipulated and utilized in an effort to achieve the primary goal of all exercise training; that is, to maximize training-induced adaptations.

## Competing interests

The authors declare that they have no competing interests.

## Authors' contributions

The comprehensive review of the literature and drafting of both the manuscript and tables was carried out primarily by KFW. RB provided figure 1 and assisted with manuscript, as well as table preparation. Both authors read and approved the final manuscript.

## Supplementary Material

Additional file 1 **Acute Exercise and Oxidative Stress: A Tabular Representation of 30 Years of Research**. The file provided displays the results of the referenced articles in tabular format. Click here for file
