# Supplementary material for: Acute exercise and oxidative stress: a 30 year history
Source: Dyn Med. 2009 Jan 13;8:1. doi: 10.1186/1476-5918-8-1 (PMC2642810; doi:10.1186/1476-5918-8-1)
Supplement: Additional file 1 — Acute Exercise and Oxidative Stress: A Tabular Representation of 30 Years of Research. The file provided displays the results of the referenced articles in tabular format. [file 1476-5918-8-1-S1.doc]

Table 1 – Studies involving non-eccentric short to moderate duration aerobic exercise without supplementation

| **Reference** | **Subjects** | **Activity** | **Tissue** | **Marker** | **Measurement Times** | **Effect** |
| --- | --- | --- | --- | --- | --- | --- |
| **(Year)** |  |  |  |  |  | **(Relative to pre exercise)** |
| Viinikka | 10 TR M | GXT on cycle | blood | LOOH | pre,post | ↔ |
| (1984) |  |  |  |  |  |  |
| Lovlin | 6 M | GXT | blood | MDA | pre,post | ↑ |
| (1987) |  |  |  |  |  |  |
| Gohil | 8 TR M | 90 min on cycle ergometer at 65% | plasma | GSH | pre, during, 0,10,15,60min, | ↓ during exercise |
| (1988) |  | VO_2peak_ |  | GSSG | 1,2,3,4 days post | ↑ during exercise |
| Marin | 3 UT & 1 TR M | 30 min TM run | blood | TGSH | rest, post | ↔ |
| (1990) |  |  |  | GSSG |  | ↔ |
|  |  |  |  | LOOH |  | ↔ |
| Kretzschmar | 18 TR and 17 UT | GXT on cycle | blood | LPO | pre, post | ↔ in UT, ↓ in T |
| (1991) | M and W |  |  | GSH |  | ↔ in UT, ↓ in T |
|  |  |  |  | GSSG |  | ↔ in UT or T |
| Sahlin | 8 M | Cycle to exhaustion | blood | xanthine | pre, 0,10min post | ↑ |
| (1991) |  |  |  | hypoxanthine |  | ↑ |
|  |  |  |  | MDA |  | ↔ |
|  |  |  |  | TGSH |  | ↑ |
| Buczynski | 41 M | exercise at 75% VO_2max_ for 20min on cycle | platelets | MDA | pre,post | ↓ |
| (1991) |  |  |  | CAT |  | ↑ |
|  |  |  |  | SOD |  | ↑ |
|  |  |  |  | GPx |  | ↑ |
| Kanaley | 12 W | TM run, 60% VO_2max_, 90 min | blood | MDA | pre, 30,60,90 during,15min | ↔ |
| (1991) |  |  |  | GPx | post | ↔ |
|  |  |  |  | GR |  | ↔ |
|  |  |  |  | CAT |  | ↔ |
| Viguie | 11 TR M | 90 min on cycle ergometer at 65% | blood | GSH | pre, during, 15 min | ↓ during exercise |
| (1993) |  | VO_2peak_ for 3 days |  | GSSG | post | ↑ during exercise |
|  |  |  |  | T-GSH |  | ↔ |
|  |  |  |  | Vitamin C |  | ↑ 15min post |
|  |  |  |  | Vitamin E |  | ↔ |
|  |  |  |  | LOOH |  | ↔ |
|  |  |  | urine | 8-OHdG |  | ↔ |
| Maxwell | 24 M & W | 1 hr box stepping | plasma | MDA | pre, 0,60min post | ↔ |
| (1993) |  |  |  | TAS |  | ↑ 0,60min post |
| Inoue | 9 swimmers & | 90 min Swim (1500m) | blood | 8-OHdG | pre, post | ↓ post in swimmers only |
| (1993) | 9 runners, M | 15km run, 70 minutes |  | hypoxanthine |  | ↑ post swim and run |
|  |  |  |  | xanthine |  | ↑ post swim and run |
| Laires | M & W | 40 min run | blood | TBARS | pre, 3min post | ↔ |
| (1993) |  |  |  | GSH |  | ↔ |
|  |  |  |  | GSSG |  | ↔ |

***Please refer to the below list of abbreviations when viewing Table 1, as well as throughout the remaining tables.***

***Definitions:*** *M, men; W, women; TR, trained; UT, untrained; DR, downhill run; TM, treadmill; MDA, malondialdehyde; TBARS, thiobarbituric acid reactive substances; LOOH, lipid hydroperoxides; CD, conjugated dienes; oxLDL, oxidized low density lipoprotein; Lag Time-LDL, susceptibility of low density lipoprotein to in vitro oxidation; Lag Time-serum, susceptibility of serum to in vitro oxidation; CD Lag Time, lag time to conjugated diene formation; PC, protein carbonyls; 8-OHdG, 8-hydroxydeoxyguanosine; TGSH, total glutathione; GSH, reduced glutathione; GSSG, oxidized glutathione; GSSG/TGSH, ratio of oxidized to total glutathione; LMW thiols, low molecular weight thiols; RBC, erythrocyte; p-SH, plasma sulfahydryl group; ESR-electron spin resonance spectroscopy; O_2_^●-^, superoxide radical; SOD, superoxide dismutase; GPx, glutathione peroxidase; GR, glutathione reductase; CAT, catalase; RAA, reduced ascorbic acid; TEAC, trolox equivalent antioxidant capacity; ORAC, oxygen radical absorbance capacity; TAS, total antioxidant status; FRAP, ferric reducing ability of plasma; TRAP, total radical-trapping antioxidant parameter; PSC, peroxyl radical scavenging capacity of plasma; Vit C, vitamin C; Vit E, vitamin E; XO, xanthine oxidase; CoQ10, coenzyme Q10; NAC, N-acetylcysteine; ↑, significant increase from pre exercise value; ↓, significant decrease from pre exercise value; ↔, no significant change; numbers following ↑,↓,↔, represent respective time points where significant findings occurred*

Table 1 – continued

| **Reference** | **Subjects** | **Activity** | **Tissue** | **Marker** | **Measurement Times** | **Effect** |
| --- | --- | --- | --- | --- | --- | --- |
| **(Year)** |  |  |  |  |  | **(Relative to pre exercise)** |
| Hartmann | 2M & 1W | GXT and 45 min steady state run | blood | DNA damage | pre, post | ↑ GXT, ↔ w/ run |
| (1994) |  |  |  | (comet assay) |  |  |
| Camus | 8 M | Uphill TM walking (35 m@ 60% | plasma | GSH | Pre, during, post | ↔ |
| . (1994) |  | VO_2max_) |  | GSSG |  | ↔ |
| Chen | 23 W & 7 M | GXT | blood | MDA | pre, post | ↑ returned at 10min post |
| (1994) |  |  |  | SOD |  | ↑ |
| Tessier | 24 UT M | 20 minTM @ 65% VO_2max_, then 100% | blood | TGSH | pre,10min post | ↓ |
| (1995) |  | VO_2max_ until exhaustion, followed by 5 min |  | GSSG |  | ↑ |
|  |  | rest and then repeated for as long as possible |  | GPx |  | ↔ |
| Laaksonen | 13 M | cycle, 40 min, 60% VO_2max_ | blood | TBARS | pre,post | ↑ |
| (1996) |  |  |  | TGSH |  | ↔ |
|  |  |  |  | GSSG |  | ↑ |
| Niess | 6 TR M & | GXT on treadmill | blood | DNA damage | pre, 15min, 24h post | ↑ 24h post |
| (1996) |  |  |  | (Comet Assay) |  |  |
|  | 5 UT M |  |  | MDA |  | ↔ |
| Leaf | 2 M & 5 W | GXT (modified Bruce protocol) | breath | expired pentane | pre, 0,5 min post | ↑ 0h post |
| ( 1997) |  |  |  | expired ethane |  | ↑ 0h post |
|  |  |  | blood | MDA |  | ↔ |
| Sumida | 22 TR M | treadmill to exhaustion | urine | 8-OHdG | pre,1,2,3 days post | ↔ |
| (1997) | 6 UT M | cycle ergometer to exhaustion | urine | 8-OHdG | pre,1,2,3 days post | ↔ |
|  |  | 20 km run | urine | 8-OHdG | pre,1,2,3 days post | ↔ |
| Pilger | 32 TR & UT | regular running exercise | urine | 8-OHdG | rest | ↔ between TR or UT |
| (1997) |  |  |  |  |  |  |
| Nishiyama | 7 M | GXT | blood | MDA | pre, post | ↔ |
| (1998) |  |  |  | SOD |  | ↔ |
| Szczesniak | 13 TR M | GXT on TM | blood | TBARS | pre, post | ↑ |
| (1998) |  |  |  | GSH |  | ↓ |
| Wetzstein | 4 M & 8 W (TR) | TM run for 30 min, 55% (UT) or 70% | blood | LDL lag time | pre,post | ↓ when data was pooled |
| (1998) | 5 M & 6 W (UT) | (TR) VO_2peak_ |  |  |  |  |
| Ashton | 12 M | cycle ergometer to exhaustion | blood | ESR spectroscopy | pre,post | ↑ |
| (1998) |  |  |  | MDA |  | ↑ |
|  |  |  |  | TAS |  | ↔ |
| Ayres | 14 W | GXT on treadmill | blood | CD | pre, post | ↔ |
| (1998) |  |  |  | CD Lag time |  | ↓ |
| Asami | 10 TR M | GXT on cycle | blood | 8-OH-Gua | pre, post | ↓ post exercise in UT M |
| (1998) | 13 UT M |  |  |  |  | No ∆ in TR M |
| Chung | 11 W | 30 min of exercise at 75-80% VO_2max_ during | blood | MDA | pre, post | ↔ |
| (1999) |  | follicular (F) and luteal (L) phases of |  | GSH |  | ↓ in both |
|  |  | menstrual cycle |  | GSSG |  | ↑ in L phase |
|  |  |  |  | TGSH |  | ↓ in F phase |

Table 1 – Continued

| **Reference** | **Subjects** | **Activity** | **Tissue** | **Marker** | **Measurement Times** | **Effect** |
| --- | --- | --- | --- | --- | --- | --- |
| **(Year)** |  |  |  |  |  | **(Relative to pre exercise)** |
| Borsheim | 8 active M | cycle ergometer, 90 min, 58±5% VO_2max_ | blood | TBARS | pre,post | ↑ |
| (1999) |  |  |  |  |  |  |
| Laaksonen | 14 M | cycle ergometer for 40 min at 60% | blood | TBARS | pre, 2 min post | ↑ |
| (1999) |  | VO_2max_ |  | TGSH |  | ↔ |
|  |  |  |  | GSH |  | ↓ |
|  |  |  |  | GSSG |  | ↑ |
|  |  |  |  | SOD |  | ↔ |
|  |  |  |  | CAT |  | ↔ |
|  |  |  |  | GPX |  | ↔ |
| Alessio | 9 M & 3 W | GXT | blood | MDA | pre,0,1h post | ↔ |
| (2000) |  |  |  | LOOH |  | ↔ |
|  |  |  |  | PC |  | ↑ 0 post |
|  |  |  |  | ORAC |  | ↑ 0 post |
| Jimenez | 7 TR M | cycle ergometer to exhaustion | blood | MDA | pre, 0,30 min post | ↔ |
| (2000) |  |  |  | Vitamin E |  | ↑ 30min post |
|  |  |  |  | SOD | pre, 24h post | ↔ |
|  |  |  |  | GPx |  | ↔ |
| Yamada | 8 TR | GXT on TM | blood | Leukocytes | pre,0,1,2h post | ↑ (neutrophils, ↑ 2h post) |
| (2000) |  |  |  | Neutrophil O_2_^●-^ |  | ↑ 0h post |
| Miyazaki | 9 UT M | acute exer to exhaustion on cycle | blood | SOD | pre, post | ↔ |
| (2001) |  |  |  | GPX |  | ↔ |
|  |  |  |  | CAT |  | ↔ |
|  |  |  |  | Neutrophil O_2_^●-^ |  | ↑ |
|  |  |  |  | RCD |  | ↔ |
|  |  |  |  | TBARS |  | ↑ |
| Hellsten | 8 TR M | 2 bouts of cycling to exhaustion w/ 60 min | muscle | urate | pre,post both bouts | ↑ |
| (2001) |  | rest in between | blood | hypoxanthine | pre,1,2,4,9,20,30,45, | ↑ in blood |
|  |  |  |  | TGSH | 60min post | ↔ |
|  |  |  |  | allantoin |  | ↑ 1-45 post |
| Bailey | 32 M | GXT on cycle to exhaustion in normoxic or | blood | LOOH | pre,post | ↑ in both |
| (2001) |  | hypoxic conditions |  | MDA |  | ↑ in both |
| Vider | 19 TR M | GXT | blood | TBARS | pre,0,30min post | ↑ 0 post |
| (2001) |  |  |  | CD |  | ↑ 0 post |
|  |  |  |  | TGSH |  | ↑ 0,30min post |
|  |  |  |  | TAS |  | ↑ 0,30min post |
|  |  |  |  | GPx |  | ↔ |
|  |  |  |  | CAT |  | ↑ 0 post |
|  |  |  |  | SOD |  | ↔ |
| Silvestro | 10 M & W | GXT | blood | TBARS | pre, post | ↔ |
| (2002) |  |  |  |  |  |  |

Table 1 – Continued

| **Reference** | **Subjects** | **Activity** | **Tissue** | **Marker** | **Measurement Times** | **Effect** |
| --- | --- | --- | --- | --- | --- | --- |
| **(Year)** |  |  |  |  |  | **(Relative to pre exercise)** |
| Davison | 13 M | GXT on cycle | blood | MDA | pre, post | ↔ |
| (2002) |  |  |  | ESR |  | ↑ when data was pooled |
|  |  |  |  | LOOH |  | ↑ when data was pooled |
| Meijer | 26 M & W | 45 min on cycle ergometer at submax level | blood | TBARS | pre,post | ↑ |
| (2002) |  |  |  |  |  |  |
| Inayama | 6 UT F | 30-min run at ventilatory threshold | blood | RBC p-SHs | pre, 5min, 1,2,24h post | ↓ 5min, 1h post |
| (2002) |  |  |  | RBC LMW thiols |  | ↓ 5min, 1h post |
|  |  |  |  | plasma p-SHs |  | ↓ 5min, 1, 24h post |
|  |  |  |  | plasma LMW thiols |  | ↔ |
| Svensson | 15 TR M | 5 days of overload training w/ a high (67%) | muscle | GSH | pre and post exercise on day | ↓ post exercise days 1, 5 |
| (2002) |  | or low (27%) carb diet, on cycle ergometer |  | TGSH | 1 and 5 and pre day 8 | ↔ |
|  |  | followed by 2.5 days of rest |  |  |  |  |
| Tozzi-Ciancarelli | UT M | acute strenuous or moderate exercise | blood | MDA | pre,post | ↑ post strenuous exercise |
| (2002) |  | strenuous=GXT on cycle |  | LDL (lag time) |  | ↑ post strenuous exercise |
|  |  | moderate=30 min ride at 60% VO_2max_ |  | TEAC |  | ↓ post strenuous exercise |
|  |  |  |  | SOD |  | ↓ post strenuous exercise |
| Elosua | 7 M & 10 W(UT) | acute cycle ergometry for 30min at ~100% | blood | SOD | pre,0,30min,1,2,24h post | ↑ 24h post before training, |
| (2003) |  | power output pre and post training |  | GPx | pre and post training | ↑ 0 and ↓ 30,60min post before |
|  |  | (training-16 wks, 3-5days/wk, 30-50min, |  | GR |  | ↓ 30min post before training |
|  |  | 65-80% VO_2max_ on cycle) |  |  |  |  |
| Rush | 18 UT M & W | 90 min on cycle ergometer at 50% VO_2max_ | blood | 8-iso-PGF_2α_ | pre, post | ↔ |
| (2003) |  |  |  | GPX |  | ↔ |
| Quindry | 9 M | 4 sessions (GXT/ submax TM exercise for | blood | MDA | pre,0,1,2h post | ↔ |
| (2003) |  | 45-60min 10% above LT and 2 x 10% below |  | LOOH |  | ↔ |
|  |  | LT |  | Neutrophil O_2_^●-^ |  | ↑ 0h post GXT and |
|  |  |  |  |  |  | 2h post 10% above LT |
| Wilber | 19 TR M | Standardized cycle ergometer interval | blood | LOOH | pre, post | ↑ |
| (2004) |  | workout (6 x 100kj) | urine | GSH | 24h pre, 24h post | ↓ |
|  |  |  |  | MDA (urine) |  | ↔ |
|  |  |  |  | 8-OHdG (urine) |  | ↔ |
| Vincent | 28 M & W | acute aerobic exercise | blood | LOOH | pre, post | ↑ |
| ( 2004) |  |  |  | TBARS |  | ↑ |
|  |  |  |  | TAS |  | ↔ |
| Orhan | 18 TR M | cycle ergometer, 60 min, 70% VO_2max_ | urine | MDA | pre,0,1,2,3 days post | ↔ |
| (2004) |  |  |  | o,o'-dityrosine |  | ↑ 0,1days post |
|  |  |  |  | 8-OHdG |  | ↑ 0,1days post |
|  |  |  |  | Butanal |  | ↑ 0days post |
|  |  |  |  | Acetone |  | ↑ 0days post |

Table 1 – Continued

| **Reference** | **Subjects** | **Activity** | **Tissue** | **Marker** | **Measurement Times** | **Effect** |
| --- | --- | --- | --- | --- | --- | --- |
| **(Year)** |  |  |  |  |  | **(Relative to pre exercise)** |
| Fatouros | 19 M (65-78 y) | 16 wks of endurance training (3 days/wk) | blood | MDA | pre, post each GXT | ↑ |
| (2004) |  | GXT pre training, post training |  | TAS |  | ↑ |
|  |  | then another GXT after |  | GPx |  | ↑ |
|  |  | 4 months of detraining |  |  |  |  |
| Di Massimo | M & W | GXT | blood | MDA | pre,post | ↑ |
| (2004) |  |  |  | LDL (lag time) |  | ↑ |
|  |  |  |  | TEAC |  | ↓ |
| Bloomer | 10 TR M | 30 min cycle at 70% VO_2max_ | blood | PC | pre,0,1,6,24h | ↔ |
| (2005) |  | DB Squats at 70% 1RM |  | MDA |  | ↔ |
|  |  |  |  | TGSH |  | ↔ |
|  |  |  |  | GSSG |  | ↑ 0 h post |
|  |  |  |  | GSH |  | ↓ 0 h post |
|  |  |  |  | 8-OHdG |  | ↔ |
| Vincent | 7 W | GXT | blood | LOOH | pre,0h post | ↔ |
| (2005) | (postmenopausal) |  |  |  |  |  |
| Elokda | 80 UT M & W | GXT | blood | GSH | pre,0,60min post | ↓ 0 h post |
| (2005) |  |  |  | GSSG |  | ↑ 0h post |
| Nikolaidis | 9 M | 2 protocols- treadmill run at 70-75% VO_2max_ | blood | TBARS | pre, 0 post | ↑ both |
| (2006) |  | 45 min, then 90% VO_2max_ till exhaustion-long |  | PC |  | ↑ both |
|  |  | or GXT-short |  | TAS |  | ↑ |
|  |  |  |  | GSH |  | ↓ long, ↔ short |
|  |  |  |  | GSSG |  | ↑ long , ↔ short |
| Steinberg | 3M & 6W (UT) | Cycle ergometry to exhaustion | plasma | TBARS | pre,0,5,20min post | ↑0,5,20min post |
| (2006) |  |  |  | RAA | pre, 5,20min post | ↓ during/ ↑ 20min post |
|  |  |  |  | GSH |  | ↓ at 0h post |
|  |  |  |  | TAS |  | ↓ 5min post/ ↑20min post |
| Goto | 8 UT M | 30 min cycle ride at 25%, 50%, 75% VO_2max_ | blood | F_2_-isoprostanes | pre,post | ↑ only w/ 75% VO_2max_ |
| (2007) |  |  |  |  |  |  |
| Steinberg | 15 UT (6W, 9M) | GXT on cycle then 5 min cool down | blood | TBARS | pre, VO_2max_ | ↑ 5,10 min post |
| (2007) |  |  |  | RAA | 5,10,20,30min post | ↓ at VO_2max_ |
|  |  |  |  | GSH |  | ↓ 10,20 min post |
| Nikolaidis | 11 boys 11 girls | 12 x 50 meter swims at 70-75% max velocity | blood | TBARS | pre, post | ↑ |
| (2007) | (TR, 9-11y old) | w/ 1 min rest between sets |  | PC |  | ↑ |
|  |  |  |  | CAT |  | ↑ |
|  |  |  |  | TAS |  | ↑ |
|  |  |  |  | GSH |  | ↓ |
|  |  |  |  | GSSG |  | ↑ |

Table 1 – Continued

| **Reference** | **Subjects** | **Activity** | **Tissue** | **Marker** | **Measurement Times** | **Effect** |
| --- | --- | --- | --- | --- | --- | --- |
| **(Year)** |  |  |  |  |  | **(Relative to pre exercise)** |
| Shing | 8 TR M | 3 days of high-intensity cycling exercise: | blood | MDA | pre,post each day | ↑ post day 1/ |
| (2007) |  | day 1- 9 x 30sec bouts at 150% peak power | urine | TAS |  | ↑ post day 1/ |
|  |  |  |  |  |  | attenuated ↑ post day 2, 3 |
|  |  | day 2- 30 km cycle ride |  | SOD |  | ↔ |
|  |  | day 3- 30 km cycle ride |  | GPX |  | ↔ |
|  |  |  |  | Vit E |  | ↓ post day 1 |
| Lwow | 200 W | 30 min physical test on cycle ergometer | blood | TBARS | pre,0,6h post | ↑ 0,6h post |
| (2007) | (postmenopausal) |  |  |  |  |  |
| Rahnama | 20 UT M | GXT on cycle | blood | TBARS | pre, post | ↔ |
| (2007) |  |  |  | PC |  | ↔ |
| Michailidis | 11 UT M | 45 min TM run at 70-75% VO_2max_ then 90% | blood | TBARS | pre,0,.5,1,2,3,4,5, | ↑ 0-3 post (peaked 1h post) |
| (2007) |  | VO_2max_ until exhaustion or rest |  | PC | 6,8,10,24h post | ↑ .5-8h post (peaked 4h post) |
|  |  |  |  | CAT |  | ↑ 0-1h post (peaked 0h post) |
|  |  |  |  | TAS |  | ↑ 0-3h post (peaked 2h post) |
|  |  |  |  | GSH |  | ↓ 0-5h post (peak ↓ at 2h post) |
|  |  |  |  | GSSG |  | ↑ .5-4h post (peaked 2h post) |
| Bloomer | 8 M & 7 W, (TR) | cycle ergometry at 70%VO_2peak_ for 30, | blood | PC | pre, 0,30,60 min post | ↑ 0,30min post 30, 60 min rides |
| (2007) |  | 60, or 120 min on 3 separate days |  |  |  | all time points for 120min ride |
| Bloomer | 15 UT M | GXT on treadmill | blood | PC | pre, post | ↑ |
| (2007) |  |  |  | MDA |  | ↔ |
|  |  |  |  | 8-OHdG |  | ↔ |
| Magalhaes | 14 TR M | TM running | blood | MDA | pre,0,1h post | ↔ |
| (2007) |  |  |  | PC |  | ↔ |
|  |  |  |  | TGSH |  | ↔ |
|  |  |  |  | GSH |  | ↔ |
|  |  |  |  | GSSG |  | ↔ |
|  |  |  |  | TAS |  | ↔ |
| Gochman | 15 M | GXT | blood | PC | pre,post | ↑ |
| (2007) |  |  |  | LOOH |  | ↔ |
|  |  |  |  | CD |  | ↔ |

Table 2 – Studies involving non-eccentric short to moderate duration aerobic exercise with supplementation

| **Reference** | **Subjects** | **Activity** | **Tissue** | **Marker** | **Measurement** | **Effect** | **Treatment/** |
| --- | --- | --- | --- | --- | --- | --- | --- |
| **(Year)** |  |  |  |  | **Times** |  | **Treatment Effect** |
| Dillard | 4 M, 1W | Cycle ergometer exercise for 1h | breath | expired pentane | pre,during,post | ↑ (no ozone effect) | 1200IU Vit E for 14 days |
| (1978) |  | w/3 ppm ozone exposure |  |  |  |  | Vit E attenuated ↑ in pentane |
| Sumida | 21 M | GXT | blood | TBARS | pre, post | ↑ | 300mg Vit E/day for 28 days |
| (1989) |  |  |  |  |  |  | TBARS↓ post w/ treatment |
| Pincemail | 5 M | cycle ergometer, 10 min at 45%, 5 min | breath | pentane | pre,during,post | ↑ | 40mg propranolol 1h pre exercise |
| (1990) |  | at 60 and 75% VO_2max_ |  |  |  |  | treatment attenuated ↑ in pentane and |
|  |  |  |  |  |  |  | decreased pentane production at rest |
| Braun | 10 TR M | GXT on cycle ergometer | blood | MDA | pre, post | ↑ | 100mg/ day of CoQ10 or placebo 56 days |
| (1991) |  |  |  |  |  |  | no treatment effect |
| Sastre | TR M | GXT Bruce protocol | blood | GSH | pre,0,30,60min post | ↓ 30,60min post | 1g GSH + 2g Vit C/day for 7 days |
| (1992) |  |  |  | GSSG |  | ↑ 0min post | prevented ↑ in GSSG |
| Kanter | 10 TR M | 30 min TM run at 60% VO_2max_, | breath | expired pentane | pre, post | ↑ | 44 days supplementation |
| (1993) | 10 UT M | followed by 5 min run at 90% | blood | MDA |  | ↑ | 148mg Vit E+250mg Vit C+7.5mg β-carotene |
|  |  |  |  |  |  |  | attenuated MDA and pentane |
| Sen | 9 M | 30 min at aerobic threshold (AeT) | blood | GSH | pre, 2 min, 24h post | ↔ | for max cycle test only: |
| (1994) |  | 30 min at anaerobic threshold (AnaeT) |  | GSSG |  | ↑ 2min post for all | supplementation w/ N-acetylcysteine (NAC) |
|  |  | max cycle ergometer test (Max) |  | TGSH |  | ↑ 2min post AnaeT | 800mg/day for 2 days+800mg pre test |
|  |  |  |  | TBARS |  | ↑ 2min post AnaeT, AeT | attenuated increase in GSSG |
|  |  |  |  | PSC |  | ↔ | PSC ↑ preexercise after treatment |
| Hartmann | 8 M | TM exercise to exhaustion | blood | MDA | pre,0,24h post | ↔ | 3 treatments (multivitamin,12h pre and 22h |
| (1995) |  |  |  | DNA damage |  | ↑ 24h post | post test/ 800mg Vit E 12h pre and 22h post |
|  |  |  |  | (Comet assay) |  |  | / 1200mg Vit E/day for 14 days pre test) |
|  |  |  |  |  |  |  | DNA damage attenuated w/ treatments |
| Alessio | 9 TR M | 30 min TM at 80% VO_2max_ | blood | TBARS | pre, post exer | ↑ | 1g a day Vit C/for 1 day |
| (1997) |  |  |  | ORAC |  | ↔ | 1g a day Vit C/for 14 days |
|  |  |  |  |  |  |  | prevented ↑ in TBARS |
| Sumida | 14 UT M | cycle to exhaustion | blood | beta-carotene | pre,post | ↓ | N=8-30mg beta-carotene a day/ |
| (1997) |  |  |  | hypoxanthine |  | ↑ | for 30 days, N=6-Placebo |
|  |  |  |  | xanthine |  | ↑ | No effect for 8-OHdG |
|  |  |  | urine | 8-OHdG | pre,1,2,3 days post | ↔ |  |
| Ashton | 10 M | incremental exercise to exhaustion | blood | ESR | pre, post | ↑ | 1000 mg ascorbic acid, 2h pre exercise |
| (1999) |  |  |  | LH |  | ↑ | treatment prevented ↑ in all markers |
|  |  |  |  | MDA |  | ↑ |  |
| Kawai | 10 UT M | GXT | blood | Serum Vit E | pre, post | ↔ | 200mg Vit E for 7 days |
| (2000) |  |  |  | RBC Vit E |  | ↓ | Vit E ↓ post treatment in serum |
|  |  |  |  |  |  |  | w/ no change in RBC |
| Akova | 18 UT W | cycle ergometer, 15 min, 50% VO_2max_ | blood | MDA | pre,post | ↔ | 300mg Vit E/day for 2 menstrual cycles |
| (2001) |  | 3 bouts of isokinetic knee extensor to |  | SOD |  | ↓ | no treatment effect |
|  |  | exhaustion |  | GPx |  | ↓ |  |

Table 2 – Continued

| **Reference** | **Subjects** | **Activity** | **Tissue** | **Marker** | **Measurement** | **Effect** | **Treatment/** |
| --- | --- | --- | --- | --- | --- | --- | --- |
| **(Year)** |  |  |  |  | **Times** |  | **Treatment Effect** |
| Bryant | 7 TR M | 90 minute cycle ride at | blood | MDA | pre,0,24h post | ↑ 0h post | 21days of (placebo/1g Vit C/day+placebo/ |
| (2003) |  | 60-70% VO_2max_ |  |  |  |  | 1g Vit C+200 IU Vit E/ 400IU Vit E+placebo |
|  |  |  |  |  |  |  | Vit E and Vit C combined |
|  |  |  |  |  |  |  | prevented ↑ in MDA |
| Waring | 20 M & W | cycle ergometer for 20 min | blood | F_2_-isoprostanes | pre, 0,20min post | ↑ 0,20min post | 5g Uric Acid in 250 ml vehicle or placebo |
| (2003) |  |  |  | TAS |  | ↔ | alone, 20 min pre exercise |
|  |  |  |  |  |  |  | prevented increase in F_2_-isoprostanes |
| Schneider | 13 TR M | TM run to exhaustion at 110% | blood | TBARS | pre,0,15min, | ↔ | 500 IU Vit E/day for 8 days pre exercise |
| (2003) |  | anaerobic threshold |  | Vit C Radical | 1,3,24,48h post | ↔ | no treatment effect |
|  |  |  |  | Vitamin E |  | ↔ |  |
|  |  |  |  | Vitamin C |  | ↔ |  |
| Medved | 8 TR M | cycle ergometer, 70% | blood | TGSH | blood-pre,during | ↓ in muscle during, 0post, | N-acetylcysteine (NAC) during exercise |
| (2004) |  | VO_2peak_, 45 min, |  |  | 0,1,2,5,10,30min post | ↔ in blood | NAC attenuated ↓ in TGSG and GSH |
|  |  | then 90%VO_2peak_ to exhaustion | muscle | GSH | Biopsy-pre, | ↔ in muscle, |  |
|  |  |  |  |  | during, 0 post | ↓ in blood during-30min |  |
|  |  |  |  | GSSG |  | ↔ in muscle, |  |
|  |  |  |  |  |  | ↑ in blood during-30min |  |
| Goldfarb | 12 M | 30 min run at 75% VO_2max_ | blood | TBARS | pre, post | ↔ | 500 or 1000mg Vit C/day for 14 days |
| (2005) |  |  |  | PC |  | ↑ | treatment attenuated ↑ in PC |
|  |  |  |  | TGSH |  | ↓ |  |
|  |  |  |  | GSH |  | ↓ |  |
|  |  |  |  | GSSG |  | ↑ |  |
| Morillas-Ruiz | 26 TR M | cycle ergometry, 90 minutes at 70% | blood | TBARS | pre, 20min post | ↔ | High antioxidant beverage 15 min pre |
| . (2005) |  | VO_2max_ |  | PC |  | ↔ | exercise and during (black grape, raspberry |
|  |  |  | urine | 8-OHdG | 24h sample | ↑ | red currant) or placebo drink |
|  |  |  |  |  |  |  | PC ↓post exercise w/ treatment |
|  |  |  |  |  |  |  | Prevented increase in 8-OHdG |
| Watson | 17 TR | 30min TM run, 60% VO_2max_, then | blood | F_2_-isoprostanes | pre,during,0,1h post | ↑ 1h post (restricted diet) | normal diet containing foods high in |
| (2005) |  | increased intensity until exhaustion |  | TAS |  | ↓ at exhaustion/↑ 1h | antioxidants, followed by a 14 day |
|  |  | (after a normal then restricted diet) |  | Vit E |  | ↔ | diet that restricted all foods high |
|  |  |  |  | beta-carotene |  | ↑ 1h post | in antioxidants |
|  |  |  |  | GSSG |  | ↑ 0,1h post |  |
| Bloomer | 24 TR M | 30 min TM run at 80% VO_2max_ | blood | PC | pre, post | ↑ | 14 day supplementation w/ |
| (2006) | 23 TR W |  |  | MDA |  | ↔ | V: 400 IU of Vit E + 1 g Vit C |
|  |  |  |  | 8-OHdG |  | ↔ | FV: fruit, veg juice powder |
|  |  |  |  | Vit C |  | ↑ at rest in V | P:placebo |
|  |  |  |  | Vit E |  | ↑ at rest in V | V and FV attenuated ↑ in PC |

Table 2 – Continued

| **Reference** | **Subjects** | **Activity** | **Tissue** | **Marker** | **Measurement** | **Effect** | **Treatment/** |
| --- | --- | --- | --- | --- | --- | --- | --- |
| **(Year)** |  |  |  |  | **Times** |  | **Treatment Effect** |
| Lu | 16 M | GXT on treadmill | blood | MDA | post GXT pre,post | ↑ | Spirulina platensis for 21 days |
| (2006) |  |  |  | SOD | treatment | ↑ | MDA and SOD attenuated |
| Tauler | 15 TR M | GXT on cycle | blood | SOD | pre,post treatment | ↔ | 250mg Vit E, 15mg beta-carotene, for 90 |
| (2006) |  | Submax- cycle ergometry, 90min, 90% |  | CAT | pre,post exercise | ↔ | days+500mg Vit C for last 15 days or |
|  |  | VO_2max_ |  | GPx |  | ↔ | placebo |
|  |  |  |  | GR |  | ↔ | CAT and GPX were higher w/ treatment |
| Gaeini | 20 M | GXT on cycle | blood | TBARS | pre, post treatment | ↔ | 450mg Vit E/day for 56 days |
| (2006) |  |  |  | PC | pre, post GXT | ↔ | no treatment effect |
| Goldfarb | 25M & | TM run,80% VO_2max_, for 30 minutes | blood | PC | pre, post | ↑ | 400 IU Vit E+1g Vit C, Fruit and Vegetable |
| (2007) | 23W(TR) |  |  | MDA |  | ↑ | powder, or placebo/ 14 days |
|  |  |  |  | 8-OHdG |  | ↔ | treatment attenuated ↓ in GSH and |
|  |  |  |  | TGSH |  | ↔ | ↑ in GSSG and PC vs. placebo |
|  |  |  |  | GSH |  | ↓ |  |
|  |  |  |  | GSSG |  | ↑ |  |

Table 3 – Studies involving acute eccentric aerobic exercise with or without supplementation

| **Reference** | **Subjects** | **Activity** | **Tissue** | **Marker** | **Measurement Times** | **Effect** | **Treatment /** |
| --- | --- | --- | --- | --- | --- | --- | --- |
| **(Year)** |  |  |  |  |  |  | **Treatment Effect** |
| Maughan | 16 M | 45 min DR | blood | TBARS | pre,0,6,24,48,72h post | ↑ 6,24,48h post |  |
| (1989) |  |  |  |  |  |  |  |
| Meydani | 9M (22-29yr) | 45 min DR @ 75% max HR | urine | TBARS | pre,0,1,2,5,12 days post | ↑ 12 days post | 800 IU vit E or for 48 days |
| (1993) | 12 M (55-74yr) |  |  |  |  |  | ↔ in TBARS w/ treatment |
| Camus | 8 M | 35 min DR @ 60% VO_2max_ | blood | GSH | Pre, during, post | ↔ |  |
| (1994) |  |  |  | GSSG |  | ↔ |  |
|  |  |  |  | Vitamin C |  | ↓ 0 , ↔20min post |  |
| Sacheck | 105 TR W | 45 min DR @ 75% max HR | blood | MDA | pre, 0, 6 24 48 hr | ↔ |  |
| (2000) |  |  |  | CD |  | ↔ |  |
|  |  |  |  | Vitamin E |  | ↔ |  |
| Sacheck | 16 M (26.6yrs), | 45 min DR @ 75% max HR | blood | F_2_-isoprostanes | pre,0,6,24,72 | ↑ 24, 72h post | 1000IU/d Vit E for 84 days |
| (2003) | 16 M(71.1yrs) |  |  | MDA |  | ↑ 0 post | attenuated increase in isoprostanes |
|  |  |  |  | 8-OHdG |  | ↔ |  |
|  |  |  |  | ORAC |  | ↓ 72h post |  |
| Kingsley | 8 active M | 50 min DR @16.5%, 8.5 km/h | blood | LOOH | pre,0,24,48h post | ↑ | 750 mg soybean-derived phosphatidylserine |
| (2006) |  |  |  |  |  |  | for 7 days pre and 2days post exercise |
|  |  |  |  |  |  |  | No treatment effect |
| Close | 20 active M | 30 min DR @ 60% VO_2max_ | blood | MDA | pre,0,1,2,3,4,7,14days | ↑ at 3,4days post | Ascorbic Acid 1g/day pre exercise and 14days |
| (2006) |  |  |  | TGSH | post | ↔ | post |
| Sacheck | 16 M (18-35y) | 45 min DR @ 75% max HR | blood | F_2_-isoprostanes | pre,0,6,24,72h post | ↑ | prevented increase in MDA |
| (2006) | 16M (65-80y) |  |  | ORAC |  | ↓ |  |

Table 4 – Studies involving long duration aerobic exercise without supplementation

| **Reference** | **Subjects** | **Activity** | **Tissue** | **Marker** | **Measurement Times** | **Effect** |
| --- | --- | --- | --- | --- | --- | --- |
| **(Year)** |  |  |  |  |  | **(Relative to pre exercise)** |
| Kanter | TR runners | 80 km race | blood | MDA | pre, post | ↑ |
| (1988) |  |  |  |  |  |  |
| Duthie | 7 M marathoners | half-marathon | blood | CD | 48,1h pre race, 5min, 24 | ↔ |
| (1990) |  |  |  | TBARS | 48,72,120h post race | ↔ |
|  |  |  |  | TGSH |  | ↓ 5min post |
|  |  |  |  | GSH |  | ↓ 5min, 24h post |
|  |  |  |  | GSSG |  | ↔ |
|  |  |  |  | GPx |  | ↔ |
|  |  |  |  | CAT |  | ↔ |
|  |  |  |  | SOD |  | ↔ |
|  |  |  |  | Vitamin A |  | ↑ 5min post |
|  |  |  |  | Vitamin C |  | ↑ 5min post |
|  |  |  |  | Vitamin E |  | ↔ |
| Vasankari | TR M | 4 trials (ran 1km, 10km,27km, | blood | CD | pre,post | ↑ post 27km run and cycle race |
| (1995) |  | 8 days of cycling-1061km) |  | TBARS |  | ↔ |
|  |  |  |  |  |  |  |
| Sanchez-Quesada | 6 TR M | continuous 4h run | blood | LDL-CD | pre,post | ↑ |
| (1995) |  |  |  | Lag time to LDL oxidation |  | ↓ |
| Ginsburg | 26 M & 13 W | triathlon | blood | Lipid Peroxides | pre,15min post | ↔ |
| (1996) | (all TR) |  |  | susceptibility to oxidation |  | ↓ |
|  |  |  |  | Vitamin A |  | ↓ |
|  |  |  |  | Vitamin C |  | ↔ |
|  |  |  |  | Vitamin E |  | ↔ |
| Inayama | 7 moderately TR M | marathon | blood | plasma protein-bound | pre,0,24,48h post | ↓ 0-48h post |
| (1996) |  |  |  | sulfhydryl group |  |  |
|  |  |  |  | TBARS |  | ↔ |
| Poulsen | 20 M | vigorous exercise 10h/day | urine | 8-OHdG | pre,post training | ↑ |
| ( 1996) |  | for 30 days |  |  |  |  |
| Dufaux | 12 moderately TR M | 2.5h run | blood | TBARS | pre,0,1,2h post & 1, | ↔ |
| (1997) |  |  |  | GSH | 2 days post | ↓ 0 post |
|  |  |  |  | GSSG |  | ↑ 0 post |
| Marzatico | 6 Marathoners (MR) | half-marathon | blood | CD | pre, 0-48h post | ↓ 24,48h post |
| (1997) | 6 Controls (all M) |  |  | MDA |  | ↑ 0h post, ↓ 24,48h post |
|  |  |  |  | SOD |  | ↑ 0h post |
|  |  |  |  | GPx |  | ↔ |
|  |  |  |  | CAT |  | ↓ 24,48h post |
| Margaritis | 12 triathletes | Long distance triathalon | blood | TBARS | pre,20min,6,24,48, | ↔ |
| ( 1997) |  |  |  | TGSH | 96h post | ↔ |
|  |  |  |  | GSH |  | ↔ |
|  |  |  |  | GSSG |  | ↔ |
| Okamura | 10 TR distance | 8 day training camp | urine | 8-OHdG | 3 days of control, during | ↑ during camp |
| (1997) | runners | (30±3 km/day) | blood | TBARS | pre,during, post camp | ↑ post camp |

Table 4 – Continued

| **Reference** | **Subjects** | **Activity** | **Tissue** | **Marker** | **Measurement Times** | **Effect** |
| --- | --- | --- | --- | --- | --- | --- |
| **(Year)** |  |  |  |  |  | **(Relative to pre exercise)** |
| Vasankari | 30 TR M | 31 km run (N=8) | blood | LDL-CD | pre, 0h post | ↔ |
| ( 1997) |  | marathon (N=22) |  | LDL-TRAP |  | ↔ |
|  |  |  |  | Serum-CD |  | ↑ in 31 km run, ↔ in marathon |
|  |  |  |  | Serum-TRAP |  | ↑ |
|  |  |  |  | Vitamin E |  | ↑ |
| Hartmann | 3 M & 3 W (TR) | short distance triathlon | urine | 8-OHdG | pre,0,24,48,72,96,120h | ↔ |
| (1998) |  |  | blood | DNA damage | post | ↑ 1-5days post |
|  |  |  |  | (comet assay) |  |  |
| Child | 17 TR runners | simulated half-marathon | blood | MDA | pre,post | ↑ |
| (1998) |  |  |  | TEAC |  | ↑ |
| Liu | 11 TR M | marathon | blood | LDL-CD | pre,0,4days post | ↔ |
| (1999) |  |  |  | Lag time to LDL oxidation |  | ↓ 0,4 days post |
|  |  |  |  | TRAP |  | ↑ 0,4 days post |
|  |  |  |  | Vitamin C |  | ↔ |
|  |  |  |  | Vitamin E |  | ↔ |
|  |  |  |  | beta-carotene |  | ↔ |
| Case | 7 TR W | half-marathon | blood | Lag phase time of CD | pre, 5min post | ↑ |
| (1999) |  |  |  |  |  |  |
| Radak | 5 TR marathon | 4 day supramarathon race | urine | 8-OHdG | pre, post race each day | ↑ post day 1 |
| ( 2000) | runners |  |  |  |  |  |
| Childs | TR M (marathoners) | 2 self paced half-marathons, | blood | MDA | pre,post each run | ↑ post 1st marathon in pooled data |
| (2000) |  | separated by 7 days |  | TEAC |  | ↑ post each run |
| Mastaloudis | 3 W & 8 M | 50 km ultramarathon | blood | F_2_-isoprostanes | baseline, 30min pre-race | ↑mid race, 0,1h post |
| (2001) | marathon runners |  |  | Vitamin C | mid-race,0,1,24h post | ↑ mid race, 0 post, ↓ 24h post |
|  |  |  |  | Vitamin E |  | ↑ mid race, 0 post, ↓ 24h post |
| Tsai | 14 M | 42 km marathon | urine | 8-OHdG | pre,0,24,72h,1,2wks post | ↑ 0-1wk post |
| (2001) |  |  | blood | DNA damage |  | ↑ 24h-2wks post |
|  |  |  |  | (comet assay) |  |  |
|  |  |  |  | MDA |  | ↑ 0-2wks post |
| Ginsburg | 38 M & 19 W | triathalon | blood | Lag time-Serum | pre, post | ↓ only in men |
| (2001) | (TR) |  |  |  |  |  |
| Kaikkonen | 21 M & 25 W | marathon | blood | TGSH | pre,post | ↔ |
| (2002) | (TR) |  |  | TRAP |  | ↑ |
|  |  |  |  | Lag time-LDL |  | ↑ |
|  |  |  |  | Lag time-Serum |  | ↓ |
|  |  |  |  | Vitamin C |  | ↑ |
|  |  |  |  | Vitamin E |  | ↔ |
| Steensberg | 11 TR M | 2.5h run on TM, 75% VO_2max_ | blood | F_2_-isoprostanes | pre,.5,1.5h into run | ↑ 1.5 into run and 0h post |
| (2002) |  |  |  |  | 0,1,2,4,8,24h post |  |
| Almar | 8 pro cyclists | 3 wk road cycling race | urine | 8-OHdG | pre,day7,14,21/pre, | ↑ 1st day or week and no further ↑ |
| (2002) |  | & a 4 day race | blood | TGSH | 1,2,3,4days | ↔ |

Table 4 – Continued

| **Reference** | **Subjects** | **Activity** | **Tissue** | **Marker** | **Measurement Times** | **Effect** |
| --- | --- | --- | --- | --- | --- | --- |
| **(Year)** |  |  |  |  |  | **(Relative to pre exercise)** |
| Radak | 6 TR M | Budapest Marathon race | muscle | endo III | 4 days pre, 17h post | ↔ |
| (2003) |  |  |  | hOGG1 |  | ↑ |
|  |  |  |  | (DNA repair enzymes) |  |  |
| Chevion | 31 TR M | 50 & 80 km march | blood | Vitamin C | pre, 0h post | ↔ |
| (2003) |  |  |  | PC |  | ↓ |
| Palazzetti | 9 TR & 6 UT | duathlon pre and post 4 wks | blood | TBARS | pre, post training | ↑ |
| (2003) | M | of overload training |  | GSH |  | ↓ |
|  |  |  |  | GSSG |  | ↑ |
|  |  |  |  | SOD |  | ↔ |
|  |  |  |  | GPx |  | ↑ |
|  |  |  |  | TAS |  | ↑ |
| Aguilo | 8 TR M | cycle mountain stage (171km) | blood | SOD | pre,0,3,15h post | ↔ |
| (2005) |  |  |  | GPx |  | ↓ 0 post |
|  |  |  |  | CAT |  | ↑ 0 post |
|  |  |  |  | GR |  | ↑ 0 post |
|  |  |  |  | GSSG |  | ↑ 0,3h post |
|  |  |  |  | vitamin E |  | ↑ 0 post |
|  |  |  |  | beta-carotene |  | ↔ |
| Tauler | 9 M | cycling mtn stage (171.8 km) | blood | MDA | pre, 3 h post | ↑ |
| ( 2006) |  |  |  | PC (lymphocyte) |  | ↑ |
|  |  |  |  | GSH |  | ↔ |
|  |  |  |  | GSSG |  | ↑ |
|  |  |  |  | SOD |  | ↑ |
|  |  |  |  | GPX |  | ↑ |
|  |  |  |  | GR |  | ↑ |
|  |  |  |  | CAT |  | ↔ |
|  |  |  |  | Vitamin E |  | ↔ |
|  |  |  |  | beta-carotene |  | ↔ |
| Knez | 45 TR & 45 UT | Half ironman triathlon | blood | MDA | pre,post | ↑ post both |
| (2007) | M & W | Full ironman triathlon |  | GPx |  | ↓ post both |
|  |  |  |  | CAT |  | ↓ post both |
|  |  |  |  | SOD |  | ↓ post both |

Table 5 – Studies involving Long duration aerobic exercise with supplementation

| **Reference** | **Subjects** | **Activity** | **Tissue** | **Marker** | **Measurement Times** | **Effect** | **Treatment/** |
| --- | --- | --- | --- | --- | --- | --- | --- |
| **(Year)** |  |  |  |  |  | **(Relative to pre exercise)** | **Treatment Effect** |
| Cooper | 10 TR | marathon | blood | SOD | pre,post | ↔ | L-carnitine loading for 10 days |
| (1986) |  |  |  | GSH |  | ↓ | no treatment effect |
|  |  |  |  | GSSG |  | ↑ |  |
| Rokitzki | 24 TR | marathon | blood | TBARS | pre,0,24h post | ↓ 0h post | 400 IU Vit E/day + 200mg ascorbic acid/ |
| (1994) |  |  |  | Vitamin C |  | ↑ 0post, then ↓ 24h post | day for 32 days pre race |
|  |  |  |  | Vitamin E |  | ↓ 24h post | no treatment effect |
|  |  |  |  | GPx |  | ↔ |  |
|  |  |  |  | CAT |  | ↔ |  |
| Kaikkonen | 37 TR M | marathon | blood | Oxidized LDL to LDL | pre,post | ↑ | 90mg Q10 +13.5mg d-α-tocopheryl |
| (1998) |  |  |  | susceptibility to oxidation |  | ↑ | acetate/day for 21 days |
|  |  |  |  | TRAP |  | ↑ | no treatment effect |
|  |  |  |  | Vitamin C |  | ↑ |  |
|  |  |  |  | Vitamin E |  | ↔ |  |
| Itoh | 14 TR M | 6 days of running, | blood | TBARS | pre supplement, | ↔ | 1200 IU Vit E/day for 28 days+ during 6 days |
| (2000) |  | 2-3h/day, 70-85% |  |  | pre,post,1, |  | TBARS decreased over 6 days w/ treatment |
|  |  | max HR |  |  | 3wks post 6 days |  |  |
| Nieman | 28 M & F (TR) | 80 km ultramarathon | blood | F_2_-Isoprostanes | pre,32km into race, post | ↑ during and post race | 1500mg/day Vit C for 7 days pre |
| (2002) |  |  |  | LOOH |  | ↑ during and post race | race |
|  |  |  |  | Vitamin C |  | ↔ | no treatment effect |
| Dawson | 15 TR M | 21 km run x 2 separated | blood | MDA | pre,0,24h post | ↔ | 500 or 1000mg Vit C + 500 or 1000 IU |
| (2002) |  | by 8 wks |  |  |  |  | Vit E/day for 28 days |
|  |  |  |  |  |  |  | no treatment effect |
| Margaritis | 20 TR M | duathlon | blood | TBARS | pre,post | ↑ | 75ųg selenium+1000IU retinyl acetate+60mg |
| (2003) |  |  |  | GSH |  | ↓ | Vit C+15IU D-α-tocopheryl succinate/day |
|  |  |  |  | GSSG |  | ↑ | for 74 days |
|  |  |  |  | SOD |  | ↔ | exacerbated ↑ in GPx w/ treatment |
|  |  |  |  | GPx |  | ↑ |  |
| Palmer | 29 M & W (TR) | ultramarathon (80km) | blood | F_2_-isoprostane | pre, post | ↑ | carb + Vit C beverage or placebo (150 |
| (2003) |  |  |  | LOOH |  | ↑ | mg/l)- drank 750ml + 500mg Vit C pill |
|  |  |  |  |  |  |  | 30min pre race + 500 ml drinks every |
|  |  |  |  |  |  |  | hour during race |
|  |  |  |  |  |  |  | no treatment effect |
| Mcanulty | 16 TR | 3h run on TM, | blood | F_2_-Isoprostanes | pre,post | ↑ | carbohydrate beverage (6% CHO) |
| (2003) |  | 70% VO_2max_ |  | LOOH |  | ↑ | no treatment effect |
|  |  |  |  | FRAP |  | ↑ |  |
| Tauler | 16 TR M | duathlon | blood | SOD | pre,0,1h post | ↔ | Supplemented on their own w/ Vit |
| (2003) |  |  |  | CAT |  | ↓ 1h post | C (treatment group had 18.3 mg/kg |
|  |  |  |  | GPX |  | ↑ 0,1h post | CAT ↑ 0h post in treatment group |
|  |  |  |  | GR |  | ↑ 1h post |  |

Table 5 – Continued

| **Reference** | **Subjects** | **Activity** | **Tissue** | **Marker** | **Measurement Times** | **Effect** | **Treatment/** |
| --- | --- | --- | --- | --- | --- | --- | --- |
| **(Year)** |  |  |  |  |  | **(Relative to pre exercise)** | **Treatment Effect** |
| Mastaloudis | 22 M &W (TR) | 50 km ultramarathon | blood | F_2_-Isoprostanes | baseline, pre,mid,0,2h, | ↑ 0h post | 1g Vit C+300mg RRR-alpha- |
| (2004) |  |  |  | Vitamin C | 1,2,3,4,6days post | ↑ mid and 0 post race | tocopheryl acetate/day for 21 days |
|  |  |  |  | Vitamin E |  | ↓ 2,5 days post | prevented increase in isoprostanes |
| Mastaloudis | 21 M & W (TR) | 50 km ultramarathon | blood | DNA damage in | pre,mid,0,2h, | ↑ mid race | 1g Vit C+400IU Vit E/day for 6 wks |
| (2004) |  |  |  | leukocytes via | 1,2,3,4,6days post |  | Women taking supplement had lower |
|  |  |  |  | comet assay |  |  | levels 1day post vs. nonsupplemented W |
| Nieman | 38 M & W (TR) | triathlon | blood | F_2_-isoprostanes | pre,5,90min post | ↑ 5min post | 800 IU Vit E/day for 60 days |
| (2004) |  |  |  | LOOH |  | ↔ | exacerbated ↑ in isoprostanes |
|  |  |  | urine | 8-OHdG |  | ↔ |  |
| Palazzetti | 17 TR | 4 wks of overload training | blood | TBARS | pre and post race | ↑ | Antioxidant mixture (Selenium A-C-E), 2 |
| (2004) |  | 4 wks normal training, |  | GSH |  | ↓ | tablets/day for 56 days during training |
|  |  | duathlon |  | GSSG |  | ↑ | no treatment effect |
|  |  |  |  | GPx |  | ↑ |  |
| McAnulty | 38 triathletes | triathlon | blood | F_2_-isoprostanes | pre, 5,90min post | ↑ 5min post | 800 IU Vit E/day for 60 days |
| (2005) |  |  |  | FRAP |  | ↑ 5,90 min post |  |
|  |  |  |  | LOOH |  | ↔ |  |
| Traber | 11M & 11W | 50km ultramarathon | blood | F_2_-Isoprostanes | pre treatment, | ↑ 0-6 days in M/ | 300mg Vit E + 400IU RRR-α-tocophyl |
| (2006) | (TR) |  |  |  |  | 0h post in W | acetate + 1000mg Vit C/ day for 42 days |
|  |  |  |  | DNA Damage | pre,mid,0,2h post race | ↑ at mid race/ | pre race and 7 days post race |
|  |  |  |  | (comet assay) | & 1-6days post | ↓ 2 days post | prevented ↑ in isoprostanes |
| Davison | 9 TR M | cycling, 60% VO_2max_, | blood | leukocytes/neutrophils | pre,0,1h post | ↑ | 1g Vit C/day for 14 days |
| (2006) |  | 2.5h |  | TAS |  | ↑ | with no effect on TAS or MDA |
|  |  |  |  | MDA |  | ↔ |  |
| Mcanulty | 63 M & W (TR) | 100 mile run | blood | F_2_-Isoprostanes | pre,~10min post | ↔ | 600 or 1200mg ibuprofen the day before |
| (2007) |  |  | urine |  |  |  | or the day of the race, respectively |
|  |  |  |  |  |  |  | ↑ in isoprostanes w/ treatment |
| Mcanulty | 15 TR M | 2.5 h cycle ride at | blood | F2-isoprostanes | pre,0,12h post | ↑ 0h post | 6% carbohydrate beverage (60g/liter) or |
| (2007) |  | 75% VO_2max_ |  | FRAP |  | ↑ 0h post | placebo 15-30min pre exercise (12ml/kg) |
|  |  |  |  |  |  |  | attenuated ↑ in isoprostanes and |
|  |  |  |  |  |  |  | exacerbated FRAP ↑ |
| Davison | 20 M | cycle ergometer at | blood | F2-Isoprostanes | pre,0,1h post | ↑ 0,1h post | 1g Vit C+400IU Vit E/day for 28 days |
| (2007) |  | 60% VO_2max_ for 2.5 h |  | TBARS |  | ↔ | TBARS decreased 0h post w/ treatment |
|  |  |  |  | Vitamin C |  | ↔ | Vit C higher 0,1h post w/ treatment |
|  |  |  |  | PAC |  | ↔ | compared to placebo |

Table 6 – Studies involving dynamic constant external resistance exercise

| **Reference** | **Subjects** | **Activity** | **Tissue** | **Marker** | **Measurement** | **Effect** | **Treatment/** |
| --- | --- | --- | --- | --- | --- | --- | --- |
| **(Year)** |  |  |  |  | **Times** | **(Relative to pre exercise)** | **Treatment Effect** |
| Mcbride | 12 TR M | high intensity resistance exercise | blood | MDA | pre,0,6,24h post | ↑ 6,24h post | 1200IU Vit E/day for 14 days |
| (1998) |  | 8 exercises, 3 sets at 10RM load |  |  |  |  | treatment prevented increase in |
|  |  |  |  |  |  |  | MDA at 6,24h post |
| Surmen-Gur | 18 UT M | performed isokinetic exercise | blood | TBARS | pre,post | ↔ | 400 mg α-tocopherol a day, 28 days |
| (1999) |  | (20 con/ecc reps, preceded by |  | SOD |  | ↓ | prevented ↓ in SOD |
|  |  | max cycle test) |  | GPx |  | ↔ |  |
| Vina | 4 TR M | Weightlifting, 5 sets of 10, for | blood | GSH | pre, post | ↔ | allopurinol, 300mg/day orally, 7 days |
| (2000) |  | quads, pecs, bracialis for 4 days |  | GSSG |  | ↑ | (3days pre, 4 days during exercise) |
|  |  |  |  |  |  |  | allopurinol prevented ↑ in GSSG |
| Subudhi | 17 TR M | 3 sets of back squats at 125,100,75% | blood | TEAC | pre,post | ↑ |  |
| (2001) |  | of body weight for as many reps as |  | MDA |  | ↔ |  |
|  |  | possible in 30,45,60 seconds |  | LOOH |  | ↔ |  |
|  |  |  |  | PC |  | ↔ |  |
|  |  |  |  | GSH |  | ↓ |  |
|  |  |  |  | GPx |  | ↔ |  |
|  |  |  |  | SOD |  | ↓ |  |
| Volek | 10 TR M | 5 sets of 15-20 reps of squats | blood | XO | pre,0,15,30,120,180 | ↑ 0,15,180min post | L-carnitine L-tartrate, 736mg per |
| (2002) |  | at 50%1RM |  | MDA | min post | ↑ 0-180min post | capsule, 4 capsules/day, 21 days |
|  |  |  |  |  |  |  | prevented ↑ in XO and MDA |
| Avery | 18 TR M | Whole body resistance protocol | blood | MDA | pre, each day before | ↑ on days 7 and 8 | 1200 IU Vit E/day for 21 days |
| (2003) |  | (4sets, 10reps, squat, BP, bent row |  |  | session for 10 days |  | no treatment effect |
|  |  | shoulder press) 3 times |  |  |  |  |  |
| Vincent | 14 M & W | Resistance training (7 exercises, | blood | LOOH | pre, post | ↑ |  |
| (2004) |  | 3 sets at 45,60,80% 1RM for 10,15 |  | TBARS |  | ↑ |  |
|  |  | 8 reps) |  | TAS |  | ↑ |  |
| Viitala | 14UT & | High intensity total-body resistance | blood | MDA | pre,0,6h post | ↑ 6h post | 885 mg Vit E/day for 14 days |
| (2004) | 13TR | training (10RM load, 24 total sets) |  |  |  |  | no treatment effect |
| Ramel | 7 TR M & | submax resistance circuit | blood | MDA | pre, 0 | ↑ |  |
| ( 2004) | 10 UT | (10 exercises, 75%1RM) |  | CD |  | ↑ only in untrained men |  |
| Bailey | 5 M | incremental exercise test on | blood | ESR | during exercise | ↑ w/ 70,100% |  |
| (2004) |  | knee ext. machine at 25,70, |  | (PBN adducts) |  |  |  |
|  |  | 100% work rate max (increments |  | LOOH |  | ↑ w/ 70% |  |
|  |  | lasted 24 minutes) |  |  |  |  |  |

Table 6 – Continued

| **Reference** | **Subjects** | **Activity** | **Tissue** | **Marker** | **Measurement** | **Effect** | **Treatment/** |
| --- | --- | --- | --- | --- | --- | --- | --- |
| **(Year)** |  |  |  |  | **Times** | **(Relative to pre exercise)** | **Treatment Effect** |
| McAnulty | 30 TR M | 10 exercises, 4 sets, 10 reps, | blood | FRAP | pre,0,1h post | ↑ 0,1h post | carbohydrate beverage (6%) or |
| (2005) |  | 40/60% 1RM |  | F2-Isoprostanes |  | ↔ | placebo pre and during exercise |
|  |  |  |  |  |  |  | no treatment effect |
| Bloomer | 10 TR M | DB Squats at 70% 1RM | blood | PC | pre,1,6,24h | ↑ 6,24h post |  |
| (2005) |  |  |  | MDA |  | ↔ |  |
|  |  |  |  | TGSH |  | ↔ |  |
|  |  |  |  | GSSG |  | ↑ 0 h post |  |
|  |  |  |  | GSH |  | ↓ 0 h post |  |
|  |  |  |  | 8-OHdG | pre, 24 h | ↔ |  |
| Liu | 19 TR W | 1 week of intensive resistance | blood | TBARS | rest, 0,2 days post | ↑ 0 post |  |
| (2005) |  | training 80% 1RM |  | MDA |  | ↑ 0 post |  |
|  |  |  |  | GSH |  | ↔ |  |
|  |  |  |  | GPx |  | ↔ |  |
|  |  |  |  | SOD |  | ↓ 0 post |  |
|  |  |  |  | TAS |  | ↔ |  |
|  |  |  |  | Vitamin C |  | ↔ |  |
|  |  |  |  | Vitamin E |  | ↓ 0 post |  |
| Bloomer | 12 TR M | barbell squats until exhaustion | muscle | PC | 2 wks before, | ↔ |  |
| (2006) |  | at 70% 1RM |  | MDA | 24h post | ↔ |  |
| Bailey | 6 TR M | single leg extension exercise | muscle | ESR | pre,post | ↑ |  |
| (2007) |  | (2min at 50% work rate max + |  | (ubisemiquinone) |  |  |  |
|  |  | 3min at 100 % WR_Max_) |  | LOOH |  | ↑ |  |
|  |  |  |  | Vitamin E |  | ↑ |  |
|  |  |  |  | beta-carotene |  | ↑ |  |
| Rietjens | 8 UT M | 8 sets of 10 on leg press and leg | urine | F_2_-isoprostanes | pre,1day post | ↑ 1 day post |  |
| (2007) |  | extension at 75% 1RM | blood | GR | pre,during,2,50,80, | ↑ during |  |
|  |  |  |  | GSSG | 110,140min post | ↔ |  |
|  |  |  |  | TEAC |  | ↑ 50,80min post |  |
|  |  |  |  | Vitamin C |  | ↑ during, 0,110,140min post |  |
|  |  |  |  | Vitamin E |  | ↑ during |  |
| Guzel | 20 UT M | High or low intensity resistance | blood | TBARS | pre,0,6,24,48, | ↑ 0h post |  |
| (2007) |  | exercise 4 exercises, 3sets, at |  |  | 72h post | (↑more w/ high intensity) |  |
|  |  | 80-95% 1RM-high or 20-35% of |  |  |  |  |  |
|  |  | high intensity load-low |  |  |  |  |  |
| Hoffman | 11 TR M | 4 sets of squats at high/low | blood | MDA | pre,0,20,40 min post | ↑ 0h post |  |
| (2007) |  | intensity (5 x 4 at 90%-high/ 5 x 15 |  |  |  |  |  |
|  |  | at 60%-low) |  |  |  |  |  |
| Bloomer | 13 TR M | (2 visits) 1) barbell squat- 1set, | blood | MDA | pre, 1 min post | ↔ |  |
| (2007) |  | 15reps, 70%1RM 2) 30 sec |  | PC |  | ↑ |  |
|  |  | max cycle sprint |  | 8-OHdG |  | ↔ |  |

Table 7 – Studies involving eccentric resistance exercise

| **Reference** | **Subjects** | **Activity** | **Tissue** | **Marker** | **Measurement Times** | **Effect** | **Treatment/** |
| --- | --- | --- | --- | --- | --- | --- | --- |
| **(Year)** |  |  |  |  |  |  | **Treatment Effect** |
| Saxton | 14 UT M | 70 eccentric & 70 concentric actions | blood | TBARS | pre,1,2,3,4,7,10days | ↔ |  |
| (1994) |  | w/ different arms (elbow flexion)- 8 |  | CD | post | ↔ |  |
|  |  | subjects | muscle | MDA | pre,0,2days post | ↔ |  |
|  |  | 80 eccentric & 80 concentric actions |  | PC |  | ↑ 0 post concentric |  |
|  |  | (knee extension)-6 subjects |  |  |  |  |  |
| Hellsten | 7 UT M | 5 x 5 minute bouts of eccentric | muscle | MDA | pre, 45min,24,48,96h | ↔ |  |
| ( 1997) |  | exercise on cycle ergometer | blood | XO | 0,15,45,90min,24,48, | ↑ 24,48,96h post |  |
|  |  |  |  | TAS | 96 h post (blood) | ↔ |  |
| Childs | 8 M & W | single leg isokinetic knee extension, | blood | MDA | pre,3,4,5,6,7,10,12 | ↔ |  |
| (1999) | (UT) | 70 max voluntary eccentric actions | muscle | TAS | days post | ↑ 4,7 days post muscle |  |
|  |  |  |  |  | biopsy: 7 days pre, |  |  |
|  |  |  |  |  | 4,7 days post |  |  |
| Radak | 12 UT W | 200 eccentric contractions of | muscle | 8-OHdG | pre,24h post | ↑ |  |
| (1999) |  | Rectus Femoris (10reps x 20sets) |  |  |  |  |  |
|  |  | 60% MIF |  |  |  |  |  |
| Childs | 14 UT M | 3set x 10reps @ 80%1RM | blood | LOOH | pre,2,3,4,7days post | ↑ 2,3,4 days post | 12.5 mg/kg Vit C and 10 mg/kg of |
| (2001) |  | (eccentric) arm curl |  | F_2_-isoprostanes |  | ↑ 3,4 days post | NAC immediately post exercise |
|  |  |  |  | SOD |  | ↑ 3 days post | for 7 days |
|  |  |  |  | GPx |  | ↔ | Markers of oxidative stress were ↑ |
|  |  |  |  |  |  |  | in supplemented group v. placebo |
| Lee | 8 M | 60 eccentric contractions at | blood | PC | pre,0,24,48,72,96h | ↑ 24,48h post |  |
| ( 2002) |  | 135-180% MIF in nondominant |  | GSH | post | ↔ |  |
|  |  | arm elbow flexor |  | GSSG |  | ↔ |  |
|  |  |  |  | TGSH |  | ↔ |  |
| Lenn | 22 M & W | 50 isokinetic eccentric elbow flexion | blood | MDA | pre supplement, pre, | ↔ | 1.8g omega-3 fatty acids/ 120 mg |
| (2002) |  | contractions |  | TBARS | 2,24,48,72h post | ↔ | soy isolate (all groups also received |
|  |  |  |  |  |  |  | 100 IU Vit E) |
|  |  |  |  |  |  |  | no treatment effect |
| Lee | 17 M | 50 max eccentric actions of elbow | blood | TGSH | rest,24,48,72,96,120 | ↑ 48-120h post |  |
| ( 2003) |  | flexors |  |  | h post |  |  |
| Goldfarb | 18 UT W | eccentric elbow flexion resistance | blood | PC | pre,0,2,6,24,48h post | ↑ 24,48h post | 400 iu Vit E, 1g Vit C, 90 ug Sel |
| ( 2005) |  | training 4sets x 12 reps |  | MDA |  | ↑ 48h post | per day/ 14days pre,2 days post |
|  |  |  |  | TGSH |  | ↓ 0,2h post | exercise |
|  |  |  |  | GSSG |  | ↑ 0,2h post | attenuated ↑ in PC and |
|  |  |  |  | GSH |  | ↓ 0,2h post | MDA vs. control |
| Bryer | 18 M | 70 eccentric elbow extensions w/ | blood | GSSG/TGSH | pre,0,4,24h post | ↑ 4,24h post | 3g Vit C/day for 14 days prior |
| (2006) |  | nondominant arm |  |  |  |  | to exercise and 4 days post |
|  |  |  |  |  |  |  | prevented↑ in GSH oxidation |

Table 7 – Continued

| **Reference** | **Subjects** | **Activity** | **Tissue** | **Marker** | **Measurement Times** | **Effect** | **Treatment/** |
| --- | --- | --- | --- | --- | --- | --- | --- |
| **(Year)** |  |  |  |  |  |  | **Treatment Effect** |
| Nikolaidis | 12 TR W | 5 x 15 max isokinetic lengthening | blood | GSH | pre,0,1,2,3,4,7days post | ↓ 2,3,4 days post |  |
| (2007) |  | knee flexions performed twice |  | GSSG |  | ↑ 2,3 days post |  |
|  |  | (2nd bout was 3 wks post 1st bout) |  | TBARS |  | ↑ 2,3,4 days post |  |
|  |  |  |  | PC |  | ↑ 2,3,4 days post |  |
|  |  |  |  | CAT |  | ↑ 2,3,4 days post |  |
|  |  |  |  | TAS |  | ↑ 3 days post |  |
| Paschalis | 10 W | eccentric knee extension | blood | TBARS | pre,24,48,72h post | ↑ 24h post |  |
| (2007) |  |  |  | PC |  | ↑ 24,48h post |  |
|  |  |  |  | GSH |  | ↓ 24,48h post |  |
|  |  |  |  | GSSG |  | ↑ 24,48h post |  |
|  |  |  |  | TAS |  | ↑ 24,48h post |  |
|  |  |  |  | CAT |  | ↑ 24,48h post |  |
| Bloomer | 36 TR M | eccentric bench press | blood | PC | pre,0,24,48h post | ↔ | 1g vit C+378mg mixed tocopherols |
| (2007) |  | (10 sets of 10 reps at 70% |  | LOOH |  | ↔ | for 14 days |
|  |  | concentric 1RM) |  |  |  |  | no treatment effect |

Table 8 – Studies involving isometric exercise

| **Reference** | **Subjects** | **Activity** | **Tissue** | **Marker** | **Measurement Times** | **Effect** | **Treatment/** |
| --- | --- | --- | --- | --- | --- | --- | --- |
| **(Year)** |  |  |  |  |  | **(Relative to pre exercise)** | **Treatment Effect** |
| Sahlin | 7 M | static knee extension exercise, | muscle | TGSH | pre, 20 min into exer, | ↑ in blood, ↔ in muscle |  |
| (1992) |  | 30%MVC for 80 min (10 sec on/ | blood | MDA | pre,during exercise | ↔ |  |
|  |  | 10 sec off) |  |  |  |  |  |
| Alessio | 9M & 3W | 50% MVC on handgrip | blood | MDA | pre,0,1h post | ↔ |  |
| (2000) |  | dynamometer, 45 sec bouts for |  | LOOH |  | ↑ 0,1h post |  |
|  |  | same duration as GXT |  | PC |  | ↔ |  |
|  |  | (GXT performed on separate day) |  | ORAC |  | ↔ |  |
| Steinberg | 7 M | 1 sec handgrip contractions for | blood | TBARS | pre,0,5,10,20,30 min | ↑ 0,5,10min post |  |
| (2002) |  | 3 min total (30 contractions per min) |  | GSH | post exercise | ↓ 0,5,10,20min post |  |
|  |  |  |  | RAA |  | ↓ 0,5,10min post |  |
| Dousset | 8 M | handgrip at 60%MVC until fatigue | blood | TBARS | pre,0,20min post | ↑ 0,20min post |  |
| ( 2002) |  | in normoxic and hypoxic conditions |  | RAA |  | ↓ 0post, then ↑ 20min post |  |
| Rodriguez | 8M & 7W | 6 cycles of max handgrip exercise | blood | MDA | pre,0,1,3,10min post | ↑ 0,1 minute post |  |
| (2003) |  | while wearing a BP cuff (30mm HG |  |  |  |  |  |
|  |  | >systolic bp) 9sec on/1 sec off for |  |  |  |  |  |
|  |  | 60 sec |  |  |  |  |  |
| Matuszczak | 10M & 8W | Left hand-handgrip at MVC until | blood | GSSG | pre, post | ↑ | 100ml solution containing NAC |
| (2005) |  | exhaustion/ Right hand- handgrip |  |  |  |  | N-acetylcysteine or saline |
|  |  | at 50%MVC (3sec on/3 sec off) |  |  |  |  | prevented GSSG ↑ |
| Sahlin | 10 active M | 5 sets static knee extension, | muscle | TBARS | pre,20min,24h post | ↔ |  |
| (2006) |  | 10 min rest between, 66% MVC |  | HNEprotein adducts |  | ↔ |  |
| Steinberg | 3M & 6W | isometric thumb adduction and handgrip | blood | TBARS | pre,during,5,20min post | ↑ 0,5min post |  |
| (2006) |  | sustained until exhaustion-50% MVC |  | RAA | pre, 5,20min post | ↓ 0,5min post |  |
|  |  |  |  | GSH |  | ↓ 0,5min post |  |
|  |  |  |  | TAS |  | ↔ |  |
| Delliaux | 13 M & W | isometric thumb adduction and handgrip | blood | TBARS | pre,post | ↑ |  |
| (2007) |  | sustained until exhaustion-50% MVC |  | RAA |  | ↑ |  |

Table 9 – Studies involving sprinting/jumping exercise

| **Reference** | **Subjects** | **Activity** | **Tissue** | **Marker** | **Measurement Times** | **Effect** | **Treatment/** |
| --- | --- | --- | --- | --- | --- | --- | --- |
| **(Year)** |  |  |  |  |  | **(Relative to pre exercise)** | **Treatment Effect** |
| Westing | 11 TR M | 800m Run | blood | hypoxanthine | pre-warm up, pre-run, 0- | ↑ 0 post |  |
| (1989) |  |  |  |  | 24h post |  |  |
| Schiffl | 3M & 3W | 2 exhaustive sprint tests, 10 min rest | blood | # of micronuclei in | pre,24,48h post | ↑ 24,48h post |  |
| (1997) |  |  |  | 3000 binucleated |  |  |  |
|  |  |  |  | blood lymphocytes |  |  |  |
| Ortenblad | 8 TR M | 6 bouts of 30sec jumps (2 min rest) | muscle | MDA | 1 wk before/ 24 hr post | ↔ |  |
| (1997) | 8UT M |  | blood | Vitamin E | pre, 0,1,2,24 hrs post | ↔ |  |
| Marzatico | 6 TR M | 6 x 150m sprints | blood | CD | pre,30min, 6,12,24,48h post | ↑ 6h post |  |
| (1997) |  |  |  | MDA |  | ↑ 6,12,24,48h post |  |
|  |  |  |  | SOD |  | ↑ 0 post |  |
|  |  |  |  | GPx |  | ↑ 0 post |  |
|  |  |  |  | CAT |  | ↔ |  |
| Svensson | 17 TR M | intermittent max cycle sprint test | blood | MDA | pre,0,20,50min post | ↔ | 120 mg of Q10 for 20 days |
| (1999) |  |  |  |  |  |  | no treatment effect |
| Thompson | 16 active M | 90 min intermittent shuttle-running test | blood | MDA | pre, 0, 24, 48, 72 | ↑ 0, 24h post | 1 g Vitamin C/day for 35 days |
| (2001) |  |  |  |  |  |  | Prevented 24h increase in MDA |
| Thompson | 9 TR M | 90 min intermittent shuttle-running test | blood | MDA | pre,post | ↑ | 1g Vit C 2h before exercise |
| (2001) |  |  |  |  |  |  | no treatment effect |
| Inal | 19 M & W | 100m swim N=9 | blood | CAT | pre,0,20,40min post | ↑ 0 post, ↓ 20,40min post |  |
| (2001) | (swimmers) | 800m swim N=10 |  | GPx |  | ↑ 0 post, ↓ 20,40min post |  |
|  |  |  |  | GSH |  | ↓ 0 post, ↑ 20,40min post |  |
| Thompson | 16 active M | Loughborough Intermittent Shuttle Test | blood | MDA | pre,0,1,2,24,48,72h post | ↑ 0,1,2,24h post | 5 day diet containing 100 mg |
| (2003) |  |  |  |  |  |  | Vit C/day + treatment group |
|  |  |  |  |  |  |  | received 400mg Vit C extra/day |
|  |  |  |  |  |  |  | for 3 days post exercise |
|  |  |  |  |  |  |  | No treatment effect for MDA |
| Groussard | 8 active M | 30 sec Wingate test | blood | TBARS | rest, 0,5,10,20,40min post | ↓ 20,40min post |  |
| (2003) |  |  |  | ESR spectroscopy |  | ↑ 20min post |  |
|  |  |  |  | GSH |  | ↔ |  |
|  |  |  |  | GPx |  | ↔ |  |
|  |  |  |  | SOD |  | ↓ 0min post |  |
| Baker | 18 M | 30-s max cycle sprint against resistive | blood | LH | rest, 0,24h post | ↑ 0 post w/ TBM protocol |  |
| (2004) |  | forces equal to total-body mass |  | MDA |  | ↑ 0 post w/ TBM protocol |  |
|  |  | (TBM) or fat-free mass (FFM) |  | Vitamin E |  | ↔ |  |
| Bloomer | 12 TR M | six, 10-s max sprints on cycle | muscle | PC | 2 wks before, 24h post | ↔ |  |
| (2006) |  |  |  | MDA | each session | ↔ |  |

Table 9 – Continued

| **Reference** | **Subjects** | **Activity** | **Tissue** | **Marker** | **Measurement Times** | **Effect** | **Treatment/** |
| --- | --- | --- | --- | --- | --- | --- | --- |
| **(Year)** |  |  |  |  |  | **(Relative to pre exercise)** | **Treatment Effect** |
| Bloomer | 13 TR M | 30 sec max cycle sprint | blood | MDA | pre, 1 min post | ↔ |  |
| (2007) |  |  |  | PC |  | ↑ |  |
|  |  |  |  | 8-OHdG |  | ↔ |  |
| Thomas | 9 TR & UT M | 1 min all out sprint on cycle | blood | TBARS | pre,0,30,60min, 24h post | ↔ | amino acid, antioxidant supplement |
| (2007) |  |  | muscle | TEAC | pre,24h post | ↔ | given for 28 days |
|  |  |  |  | GPx |  | ↔ | ↓ TBARS and↑ GPx |
|  |  |  |  |  |  |  | activity at rest |

Table 10 – Studies involving a variety of sporting events

| **Reference** | **Subjects** | **Activity** | **Tissue** | **Marker** | **Measurement Times** | **Effect** |
| --- | --- | --- | --- | --- | --- | --- |
| **(Year)** |  |  |  |  |  | **(Relative to pre exercise)** |
| Subudhi | 12 elite alpine skiers | On-snow slalom and giant slalom | blood | TEAC | pre,post | ↔ |
| (2001) |  | training (15 runs) |  | MDA |  | ↔ |
|  |  |  |  | LOOH |  | ↓ |
|  |  |  |  | PC |  | ↔ |
|  |  |  |  | GSH |  | ↔ |
|  |  |  |  | GPx |  | ↔ |
|  |  |  |  | SOD |  | ↓ |
| Schroder | 13 M, pro basketball players | 8-10 basketball related training sessions | blood | LOOH | pre treatment, training, | ↓ 24h post training |
| (2001) |  | ~90 min, 2days/wk for 35 days |  | Vitamin C | 0,24h post training | ↔ |
|  |  |  |  | Vitamin E |  | ↔ |
|  |  |  |  | Beta-carotene |  | ↓ 24h post training |
| Schippinger | 8 pro football players | training + competition, 4 sessions/wk | blood | Total Peroxides | 4 time points (pre#1, | ↑ at #4 compared to #1, #2 |
| (2002) |  | for 2h and 9 games |  | Lipid perox Lag time | post 3 different games, | ↔ |
|  |  |  |  | OxLDL antibodies | #2,#3,#4) | ↑ at #3 compared to #1 |
| Chang | 6 weekend warriors (vigorous | rugby game | blood | CD | pre, post game | ↑ |
| (2002) | exercise once/wk-WW) & 15 |  |  | TBARS |  | ↑ |
|  | trained rugby players-TR & 10 |  |  |  |  |  |
|  | sedentary controls-CON (M) |  |  |  |  |  |
| Evelson | 15 rugby players & 15 sedentary | regular training for rugby season | blood | TBARS | fasted sample | ↔ between groups |
| (2002) | M |  |  | TRAP |  | ↑ in rugby players |
|  |  |  |  | SOD |  | ↑ in rugby players |
|  |  |  |  | CAT |  | ↔ between groups |
| Metin | soccer players & controls, M | regular soccer training | blood | MDA | at rest | ↓ vs. controls |
| (2003) |  |  |  | plasma thiols |  | ↑ vs. controls |
| Kingsley | 16 M, soccer players | exhaustive intermittent exercise (90min) | blood | LOOH | pre, 15min, 24, 48h post | ↑ 15min post |
| (2005) |  |  |  | CD Lag time |  | ↑ 15min,24,48h post |
|  |  |  |  | Vitamin C |  | ↑ 15min post |
|  |  |  |  | Vitamin E |  | ↔ |
|  |  |  |  | Beta-carotene |  | ↔ |
| Magalhaes | 14 trained climbers, M | continuous climbing until exhaustion | blood | MDA | pre,0,1h post | ↑ 0,1h post |
| (2007) |  | TM running at same intensity |  | PC |  | ↑ 0h post |
|  |  | and duration as climb |  | TGSH |  | ↓0,1h post |
|  |  |  |  | GSH |  | ↓ 0,1h post |
|  |  |  |  | GSSG |  | ↑ 0,1h post |
|  |  |  |  | TAS |  | ↑ 0h post |
| Ascensao | 10 M, pro motocross | simulated competitive | blood | TGSH | pre,0,1h post | ↓ 0,1h post |
| (2007) | athletes | motocross race |  | GSH |  | ↓ 0,1h post |
|  |  |  |  | GSSG |  | ↑ 0,1h post |
|  |  |  |  | MDA |  | ↑ 0h post |
|  |  |  |  | PC |  | ↑ 0,1h post |
|  |  |  |  | TAS |  | ↑ 0,1h post |
